# Phylogeny and biogeography of the African Bathyergidae: a review of patterns and processes

**DOI:** 10.7717/peerj.7730

**Published:** 2019-10-15

**Authors:** Jacobus H. Visser, Nigel C. Bennett, Bettine Jansen van Vuuren

**Affiliations:** 1 Centre for Ecological Genomics and Wildlife Conservation, Department of Zoology, University of Johannesburg, Johannesburg, Gauteng Province, South Africa; 2 Department of Zoology and Entomology, University of Pretoria, Pretoria, Gauteng Province, South Africa

**Keywords:** African mole-rats, Bathyergidae, Biogeography, Extra-limital, Species richness, Phylogeography

## Abstract

**Background:**

We review genealogical relationships, biogeographic patterns and broad historical drivers of speciation within the Bathyergidae, a group of endemic African rodents, as well as identify key taxa which need further research.

**Methods:**

We sourced comparable cytochrome *b* sequence data (comparable data available for all members for the Family) and geographic information for all six genera of the African subterranean rodent. This information was combined into the most comprehensive and geographically representative evolutionary study for the Bathyergidae to date.

**Results:**

Species richness within the Bathyergidae appears to be underestimated, with undescribed taxa in five of the six genera. Biogeographic patterns suggest large historical distributions, which were repeatedly fragmented by major landscape changes (especially rifting, uplift and drainage evolution) since the Miocene. Aside from vicariant events, other factors (ecological specialization, population-level responses and climatic change) may have been instrumental in driving divergences in the Bathyergidae. As such, adaptive differences may exist among both populations and species across their discrete ranges, driving independent evolutionary trajectories among taxa. In addition, highly fragmented distributions of divergent (and often relict) lineages indicates the possibility of narrow endemics restricted to diminishing suitable habitats. From this, it is clear that a systematic revision of the Bathyergidae is necessary; such a revision should include comprehensive sampling of all putative taxa, the addition of genomic information to assess adaptive differences, as well as ecological information.

## Introduction

Biodiversity is being lost at unprecedented rates, with extinction risk being underestimated for a large number of species in the absence of explicit geospatial and/or population data (Ceballos et al., 2015; Ocampo-Peñuela et al., 2016). It is further clear that species are not ubiquitously distributed across their ranges; habitat specialists are limited to areas of suitable habitat, leading to the possibility of undescribed taxa (Pimm et al., 2014). Accurate and detailed information on species’ distributions and evolutionary histories fill an important void in optimal conservation planning, with DNA evidence aiding in the uncovering of undescribed taxa, and elucidating biogeographic patterns and the drivers thereof.

**Figure 1:**
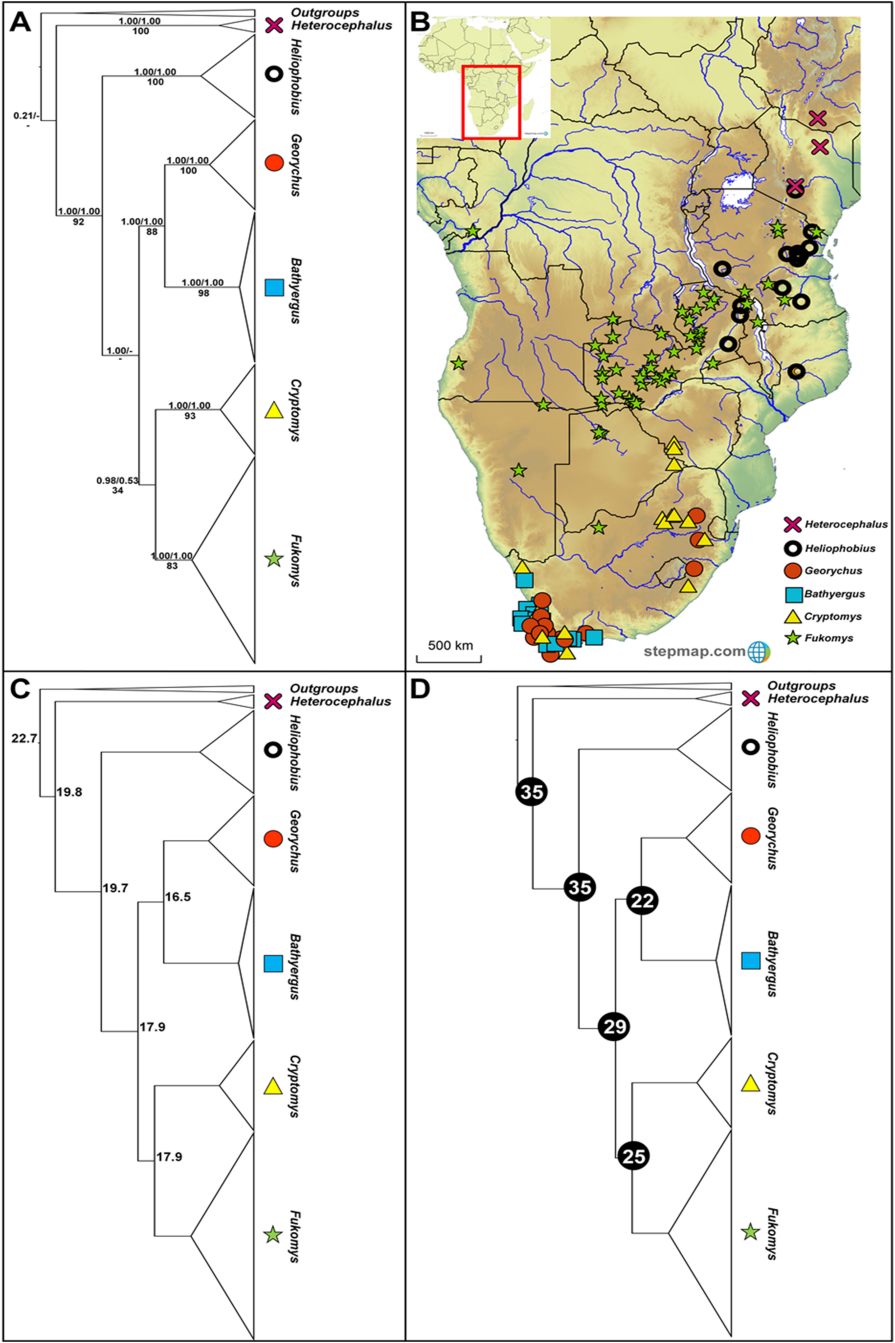
Phylogeny, geographic distributions, sequence divergences and divergence dates of the six bathyergid genera. (A) Phylogeny of all six bathyergid genera based on the cytochrome *b* data. Values above nodes represent posterior probability values derived from the Bayesian analysis in BEAST and MrBayes respectively, while values below nodes indicate bootstrap values derived after Maximum Likelihood analysis in RAxML. A “-” indicates that the node was not retrieved by the particular analysis. The geographic distribution of these genera across sub-Saharan Africa is portrayed in (B) with the uncorrected sequence divergences (%) at the various nodes shown in (C). The divergence date estimates (in Mya) for each node is indicated within the black circles in (D).

A case in hand concerns the Bathyergidae, a group of subterranean rodents endemic to sub-Saharan Africa (Faulkes et al., 2010; Ingram, Burda & Honeycutt, 2004; Honeycutt, 2017). The family currently comprises six monophyletic genera (Allard & Honeycutt, 1992; Walton, Nedbal & Honeycutt, 2000; Huchon & Douzery, 2001; Faulkes et al., 1997, 2004; Ingram, Burda & Honeycutt, 2004) with largely disjunct distributions across their ranges. *Heterocephalus* and *Heliophobius* are restricted to East Africa, *Bathyergus* and *Georychus* to South Africa, with *Cryptomys* and *Fukomys* having wide distributions across southern and south-central Africa (see Fig. 1; Honeycutt et al., 1991).

The social systems within this family range from solitary (*Heliophobius*, *Georychus* and *Bathyergus*) to eusocial (*Heterocephalus* and *Fukomys*; Ingram, Burda & Honeycutt, 2004). Because of their eusocial nature (relatively uncommon amongst mammals), *Heterocephalus* and *Fukomys* have received much attention (Bennett & Jarvis, 1988; Nevo & Reig, 1990; Sherman, Jarvis & Alexander, 1991; Jarvis & Bennett, 1993; Nevo, 1999; Bennett & Faulkes, 2000; Lacey, Patton & Cameron, 2000; Faulkes & Bennett, 2007, 2009), leaving the other genera largely under-investigated by comparison (but see Honeycutt et al., 1987; Allard & Honeycutt, 1992; Faulkes et al., 1997; Walton, Nedbal & Honeycutt, 2000; Huchon & Douzery, 2001). Recent systematic/phylogeographic studies have hinted that diversity within the family is notably under-represented (Faulkes et al., 2004; Ingram, Burda & Honeycutt, 2004; Visser, Bennett & Jansen van Vuuren, 2014, 2018, 2019) and as a result, several species have recently been described (Honeycutt et al., 1991; Aguilar, 1993; Macholan et al., 1998; Burda et al., 1999; Chitaukali, Burda & Kock, 2001; Gippoliti & Amori, 2011; Van Daele et al., 2013; Faulkes et al., 2017), with further suggestions of possibly undescribed species in *Heliophobius* (Faulkes et al., 2011), *Georychus*, *Bathyergus* and *Cryptomys* (Visser, Bennett & Jansen van Vuuren, 2019).

Given the spate of phylogeographic investigations across wide geographic areas (Faulkes et al., 2004, 2011, 2017; Van Daele et al., 2007b; Visser, Bennett & Jansen van Vuuren, 2014, 2018, 2019), sufficient information now exist to synthesize a comprehensive systematic and biogeographic review for the Bathyergidae based on comprehensive geographic coverage and including all the bathyergid genera. In doing so, we construct a comprehensive phylogeny for the group to review genealogical relationships and timing of diversification events, and identify and review biogeographic patterns and broad historical processes driving speciation. This study therefore represents the most comprehensive and geographically representative study for the Bathyergidae to date, encompassing some 740 individuals representing all six genera. We use this information to assess the integrity of current species, as well as highlight possible undescribed cryptic species. To this end, we base inferences on consistent and well-supported monophyly together with sequence divergence estimates (with reference to other intra-generic divergences). Finally, we comment on future directions for research into this group.

## Materials and Methods

### Specimens

The cytochrome *b* gene region has been the predominant marker of choice in phylogeographic investigations within the bathyergid genera, and a representative dataset of this marker is available for a large number of species and lineages within the Bathyergidae including a comprehensive coverage of geographic localities. Comparable cytochrome *b* sequence data were sourced from public databases for all six mole-rat genera (*Heterocephalus*, *Heliophobius*, *Georychus*, *Bathyergus*, *Cryptomys* and *Fukomys*, see Fig.1; Table S1). These data were aligned using ClustalW as implemented in Geneious Pro™ 7.0 software (Biomatters Ltd, Auckland, New Zealand). Sequences shorter than 735 bp were removed to ensure a dataset with minimal missing data (see Joly, Stevens & van Vuuren, 2007 for a discussion of the impact of missing data).

Because of the large sample size (see below) and to reduce computational time, analyses are based on haplotypes only; alignments of the data were deconstructed into haplotypes using TCS 1.21 (Clement, Posada & Crandall, 2000). In total, 740 sequences (see Table S2 for species names and authorities) were included (*Heterocephalus*: *n* = 7 from three localities; *Heliophobius*: *n* = 60 from 19 localities; *Georychus*: *n* = 265 from 15 localities; *Bathyergus*: *n* = 190 from 15 localities; *Cryptomys*: *n* = 76 from 22 localities; *Fukomys*: *n* = 142 from 60 localities) which collapsed into 289 haplotypes (*Heterocephalus*: *n* = 7; *Heliophobius*: *n* = 38; *Georychus*: *n* = 41; *Bathyergus*: *n* = 68; *Cryptomys*: *n* = 41; *Fukomys*: *n* = 94). In addition, outgroup taxa of successive relatedness, and representing the Hystricognathi, were included to root topologies. These included the porcupine (*Hystrix africaeaustralis*), the dassie rat (*Petromus typicus*) and the cane rat (*Thryonomys swinderianus*).

### Genealogical and molecular dating analyses

Prior to genealogical analyses, we used the Translate Tool in ExPASy (SIB Bioinformatics Resource Portal) to find the open reading frame in the data, and Split Codons (Sequence Manipulation Suite, www.Bioinformatics.org) to remove each codon position as a separate dataset (1st, 2nd and 3rd). Thereafter, the best-fit substitution model for each codon (1st position: TN93+G; 2nd position: GTR+G; 3rd position: HKY85+G+I) was selected through Modeltest version 2.1 (Guindon & Gascuel, 2003; Darriba et al., 2012) using the Akaike information criterion (Akaike, 1973). Phylogenetic reconstructions followed Bayesian inference methods and maximum likelihood (ML) with nodal support determined through posterior probabilities and bootstrapping respectively. Posterior probabilities ≥ 0.90 and bootstraps ≥ 70% were considered acceptable support.

Bayesian inference trees were constructed in MrBayes version 3.2.7 (Ronquist et al., 2012) by running 5 × 10^6^ generations with sampling every 1,000 generations. To assess adequate convergence of the Markov chain Monte Carlo (MCMC) chain, the effective sample size of parameters was verified in Tracer version 1.5 (Rambaut & Drummond, 2003). After discarding the first 25% of the trees as burnin, a majority rule consensus tree with posterior probabilities was constructed and visualized in Figtree version 1.4.2 (available at: http://tree.bio.ed.ac.uk/software/figtree/).

Maximum likelihood analyses were performed in RAxML version 7.0.4 (Stamakis, 2014), which consisted of a 1,000 bootstrap replicates followed by a ML search. A majority rule consensus was constructed and visualized using Dendroscope version 3.5.8 (Huson & Scornavacca, 2012).

To obtain estimates of divergence times for the various clades, an uncorrelated relaxed molecular clock model with a Yule tree prior (most suitable for inter-specific comparisons) and an UPGMA starting tree was adopted in BEAST version 1.6.1 (Drummond et al., 2012). A fossil calibration point for *Heliophobius* (19 ± 0.5 Mya; Lavocat, 1973) was included in the analysis. Runs were continued for 30 × 10^6^ generations with sampling every 1,000 generations. Results were visualized in Tracer version 1.5 (Rambaut & Drummond, 2003) to assess sufficient mixing and convergence of the MCMC chains, and samples from the posterior distribution of trees were summarized in TreeAnnotator version 1.6.1 (Drummond et al., 2012) with burnin set to 25%. Finally, a consensus tree was constructed using Figtree version 1.4.4 (available at: http://tree.bio.ed.ac.uk/software/figtree/).

In the final phylogenetic tree, nomenclature follows existing species (as listed on public databases) within the Bathyergidae, and the genetic affinity of sequences from unnamed specimens to the existing species (see Table S1). Possibly undescribed species are identified as lineages which are consistently retrieved as monophyletic and with acceptable nodal support (posterior probabilities ≥ 0.90 and bootstraps ≥ 70%) among the different analyses. Furthermore, these possibly undescribed species were identified on the basis of substantial sequence divergence (divergences that are higher than between described species within that genus, or the intrageneric mean). Calculation of uncorrected sequence divergences were performed in DnaSP version 5.10.01 (Librado & Rozas, 2009).

## Results

### Genealogical- and biogeographic patterns

#### Genera

The six genera form well-supported monophyletic groups in all the genealogical analyses (see Fig. 1A). *Heterocephalus* groups separately in the phylogeny relative to the remaining Bathyergidae; supporting the recognition of two subfamilies Bathyerginae and Heterocephalinae (none of our analyses recovered strong support for the monophyly of the Bathyergidae, although other genes and lines of evidence do support the monophyly of the mole-rats; see Davies et al., 2015). Within the Bathyerginae, *Heliophobius* occupies a basal position relative to the other four genera. The South African endemic genera *Georychus* and *Bathyergus* are well-supported sister genera with the sister relationship between the widespread genera *Cryptomys* and *Fukomys* retrieved, but not supported by two of the analyses (MrBayes and RAxML).

Generic ranges appear, to some extent, discrete for three of the genera (*Heterocephalus*, *Heliophobius* and *Fukomys*), but with some degree of overlap between *Heliophobius* and *Fukomys*, and extensive overlap between the southern African genera *Georychus*, *Bathyergus* and *Cryptomys* (Fig. 1B). Sequence divergences among the genera are generally in the same order of magnitude (17.7– 25.5%; Fig. 1C; Table S3) with divergences between *Georychus* and *Bathyergus*, and *Cryptomys* and *Fukomys*, largely contemporaneous during the Oligocene (Fig. 1D). Divergences among these generic complexes, and the remaining members of the Bathyergidae, appear more ancient and in the Eocene (Fig. 1D).

#### Heterocephalus

Within *Heterocephalus* no major clades are evident; however, there appears to be some separation between animals from Ethiopia and Kenya (see Fig. S1).

#### Heliophobius

**Figure 2:**
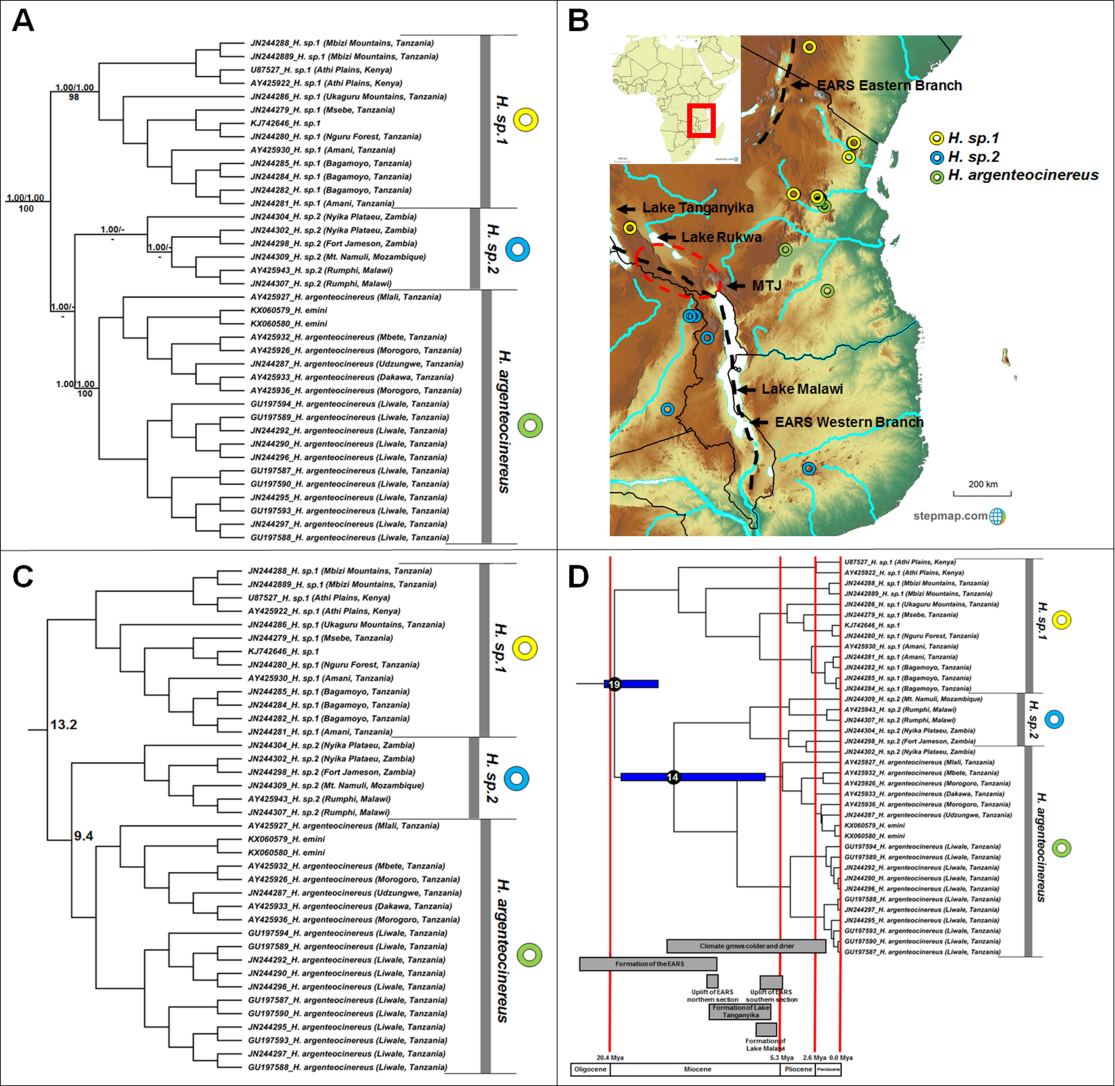
Phylogeny, geographic distributions, sequence divergences and divergence dates of species within the genus *Heliophobius*. (A) Phylogeny of the genus *Heliophobius* based on the cytochrome *b* data and indicating the different species included and identified. Values above nodes represent posterior probability values derived from the Bayesian analysis in BEAST and MrBayes respectively, while values below nodes indicate bootstrap values derived after Maximum Likelihood analysis in RAxML. A “-” indicates that the node was not retrieved by the particular analysis. The geographic location (point locality for the specimen) of the recovered species is portrayed in (B) with the uncorrected sequence divergences (%) at the various nodes shown in (C). The divergence date estimates (in Mya) for each node is indicated within the black circles in (D), with the blue bars representing the temporal range of the divergence (derived from the dating analysis in BEAST). The red bars represent the temporal boundaries among the epochs, with the timing and temporal extent of major landscape changes which occurred across the distribution of the genus indicated at the bottom.

*Heliophobius* comprises three major clades (Fig. 2A), but with some variations among the sister taxon relationships retrieved by the different analyses. In the BEAST phylogeny (shown), *H. sp. 2* is sister to *Heliophobius argenteocinereus*, while both MrBayes and RAxML groups *H. sp. 1* and *Heliophobius argenteocinereus* as sister taxa with strong support. *Heliophobius argenteocinereus* and *H. sp. 1* both occur to the east of the East African Rift System, whereas *H. sp. 2* occupies areas to the west of this region (Fig. 2B). The sister relationships between these three lineages are not clear, with sequence divergence values supporting *H. sp. 2* and *Heliophobius argenteocinereus* as closer related (9.4%; Fig. 2C; Table S4). Divergence times among the *Heliophobius* species also indicate a more recent middle Miocene divergence between *H. sp. 2* and *Heliophobius argenteocinereus* (Fig. 2D).

#### Georychus

**Figure 3:**
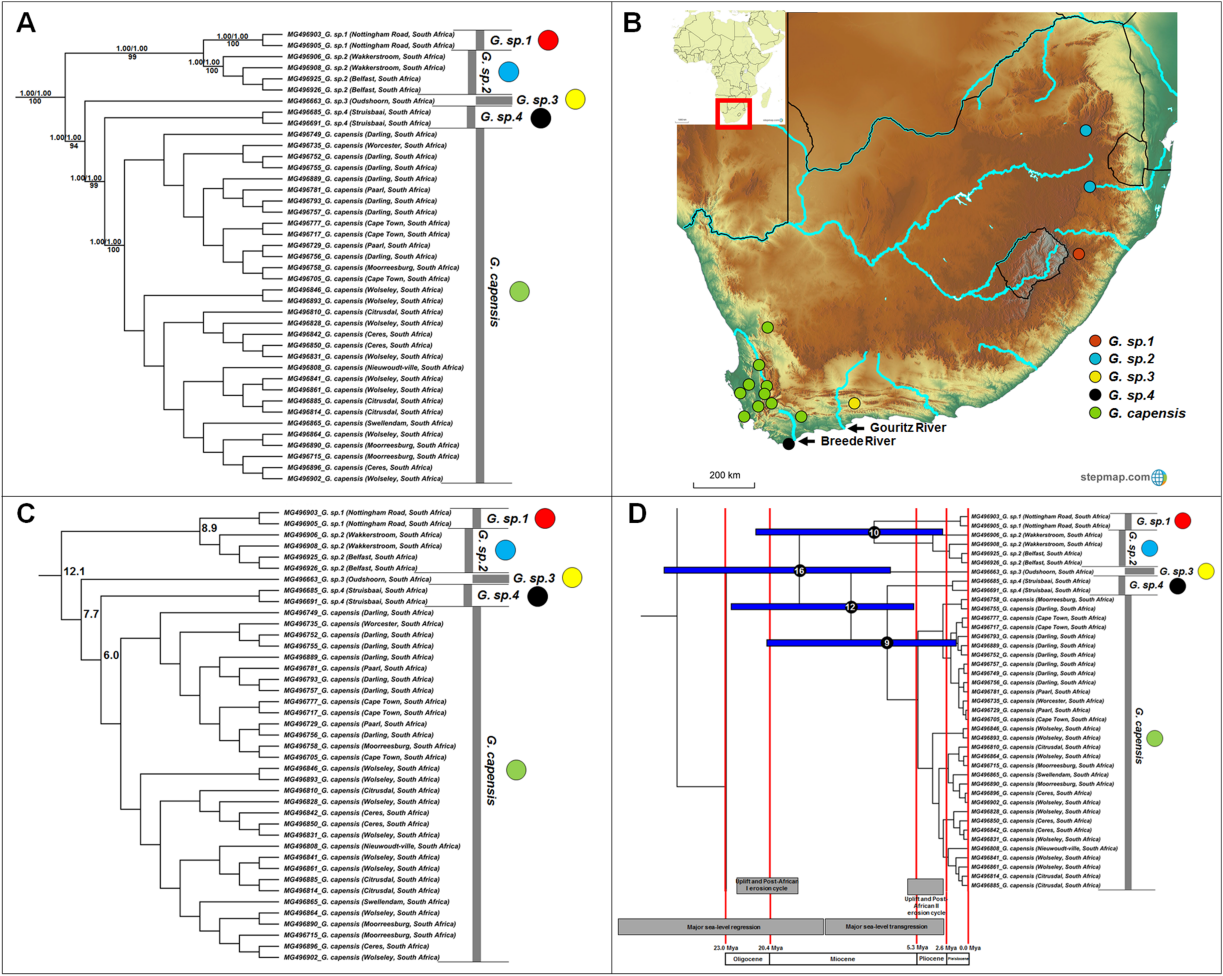
Phylogeny, geographic distributions, sequence divergences and divergence dates of species within the genus *Georychus*. (A) Phylogeny of the genus *Georychus* based on the cytochrome *b* data and indicating the different species included and identified. Values above nodes represent posterior probability values derived from the Bayesian analysis in BEAST and MrBayes respectively, while values below nodes indicate bootstrap values derived after Maximum Likelihood analysis in RAxML. The geographic location (point locality for the specimen) of the recovered species is portrayed in (B) with the uncorrected sequence divergences (%) at the various nodes shown in (C). The divergence date estimates (in Mya) for each node is indicated within the black circles in (D), with the blue bars representing the temporal range of the divergence (derived from the dating analysis in BEAST). The red bars represent the temporal boundaries among the epochs, with the timing and temporal extent of major landscape changes which occurred across the distribution of the genus indicated at the bottom.

*Georychus* includes five well-supported, geographically discreet, clades (Fig. 3A) which follow a north-east to south-west clinal pattern across South Africa (Fig. 3B). *Georychus sp. 1* and *G. sp. 2* appear highly divergent from each other (8.9%) in spite of their geographic proximity (Mpumalanga and KwaZulu-Natal provinces, both in the north eastern part of South Africa), as well as from their congenerics in the Western Cape Province of South Africa (9.9–12.5%; Fig. 3C; Table S5). The times of divergences among the five species span the Miocene (Fig. 3D).

#### Bathyergus

**Figure 4:**
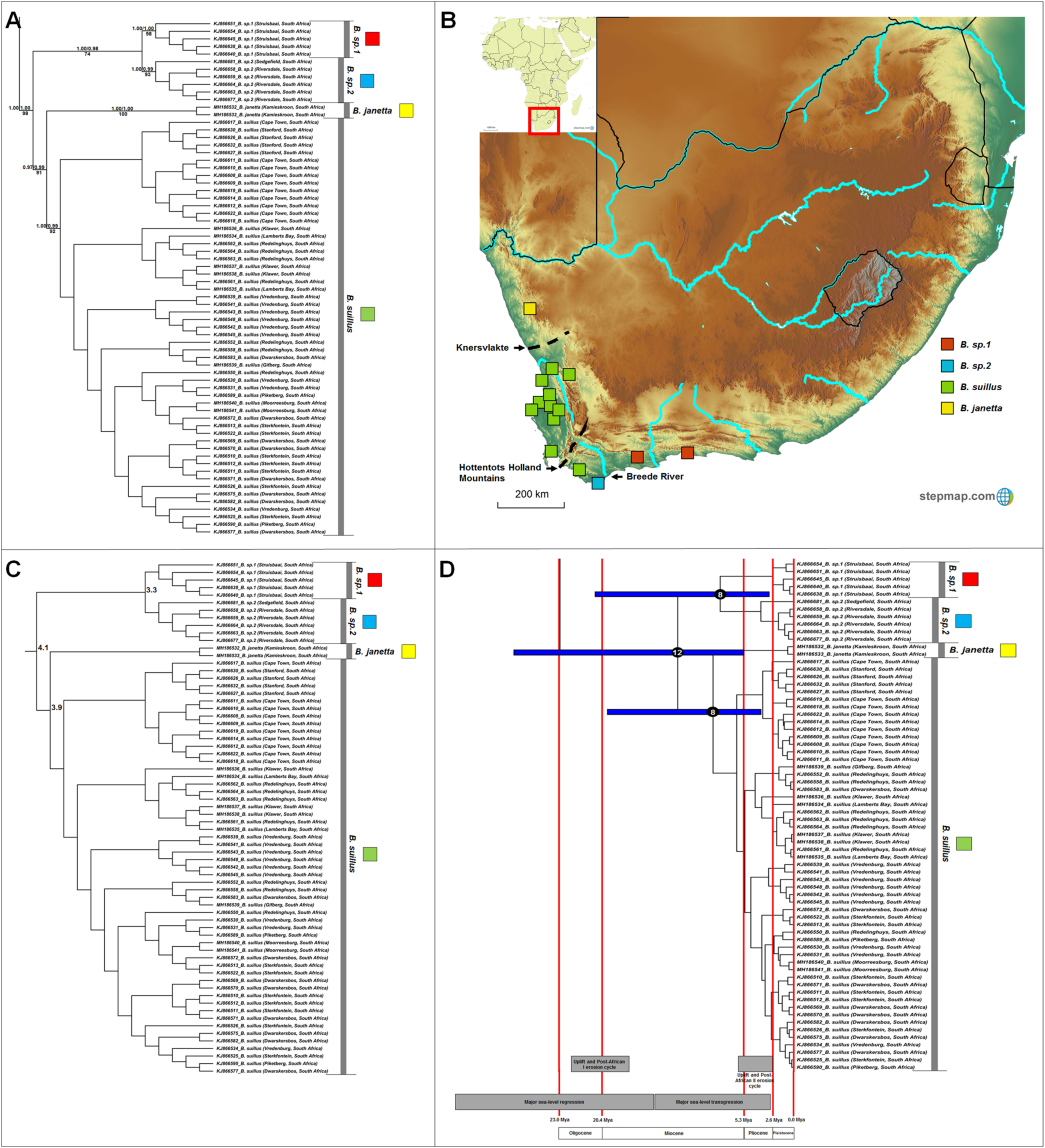
Phylogeny, geographic distributions, sequence divergences and divergence dates of species within the genus *Bathyergus*. (A) Phylogeny of the genus *Bathyergus* based on the cytochrome *b* data and indicating the different species included and identified. Values above nodes represent posterior probability values derived from the Bayesian analysis in BEAST and MrBayes respectively, while values below nodes indicate bootstrap values derived after Maximum Likelihood analysis in RAxML. The geographic location (point locality for the specimen) of the recovered species is portrayed in (B) with the uncorrected sequence divergences (%) at the various nodes shown in (C). The divergence date estimates (in Mya) for each node is indicated within the black circles in (D), with the blue bars representing the temporal range of the divergence (derived from the dating analysis in BEAST). The red bars represent the temporal boundaries among the epochs, with the timing and temporal extent of major landscape changes which occurred across the distribution of the genus indicated at the bottom.

Four well-supported major clades are evident within *Bathyergus* (Fig. 4A) which occupy non-overlapping ranges across South Africa (Fig. 4B). Sequence divergence values between major clades within this genus is lower compared to those seen in the other genera (3.3–5.3%; Fig. 4C; Table S6), resulting is slightly more recent divergence date estimates (Fig. 4D).

#### Cryptomys

**Figure 5:**
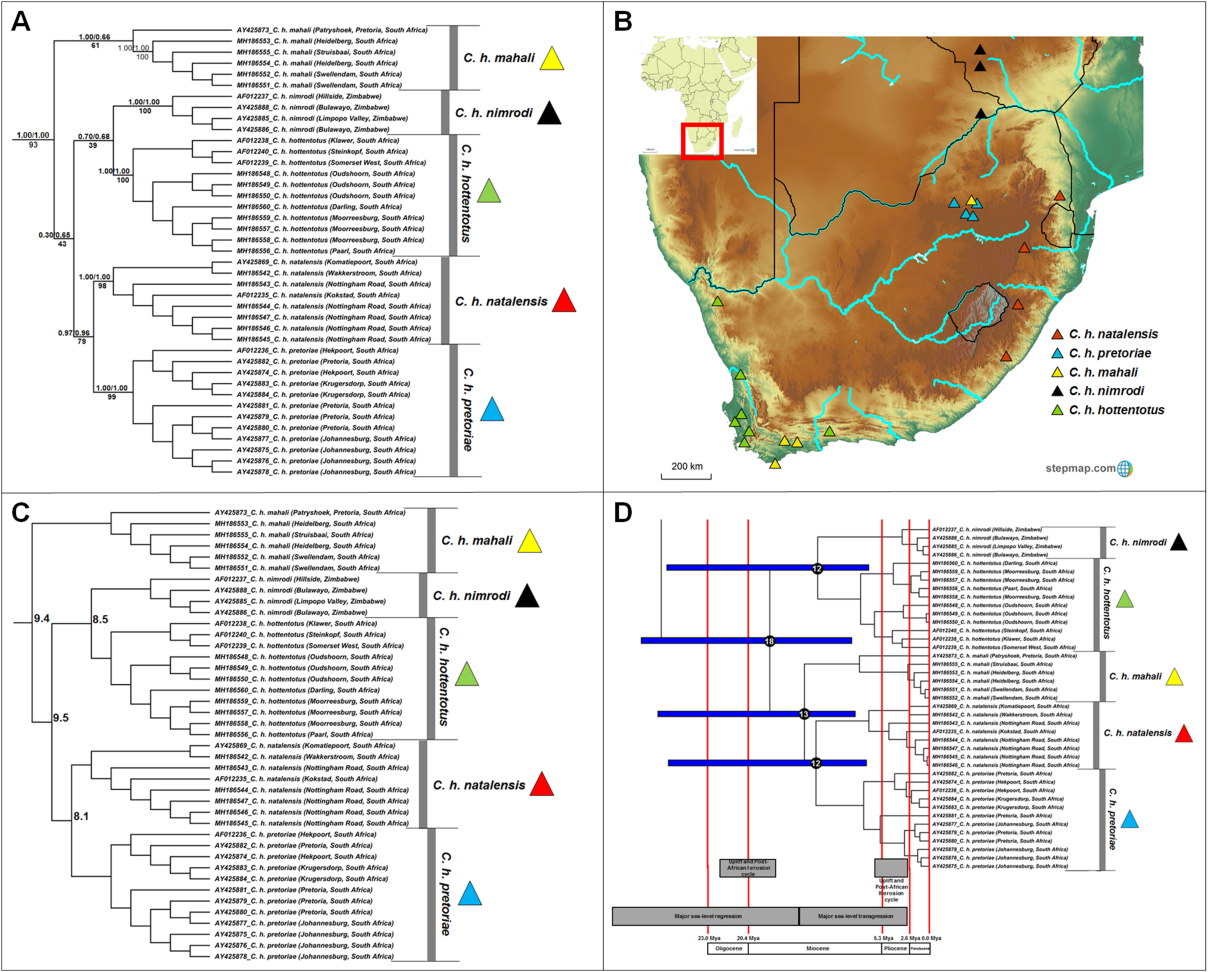
Phylogeny, geographic distributions, sequence divergences and divergence dates of species within the genus *Cryptomys*. (A) Phylogeny of the genus *Cryptomys* based on the cytochrome *b* data and indicating the different species included and identified. Values above nodes represent posterior probability values derived from the Bayesian analysis in BEAST and MrBayes respectively, while values below nodes indicate bootstrap values derived after Maximum Likelihood analysis in RAxML. Values in normal font show support for species which were recovered with the exclusion of a particular specimen. The geographic location (point locality for the specimen) of the recovered species is portrayed in (B) with the uncorrected sequence divergences (%) at the various nodes shown in (C). The divergence date estimates (in Mya) for each node is indicated within the black circles in (D), with the blue bars representing the temporal range of the divergence (derived from the dating analysis in BEAST). The red bars represent the temporal boundaries among the epochs, with the timing and temporal extent of major landscape changes which occurred across the distribution of the genus indicated at the bottom.

Five major clades, which corresponds to the described subspecies, were retrieved within *Cryptomys* (Fig. 5A); four of these clades received strong nodal support from all genealogical analyses (*Cryptomys h. nimrodi*, *C. h. natalensis*, *C. h. pretoriae* and *C. h. hottentotus*), with the exception of *C. h. mahali* which was only well-supported in one analysis (BEAST). The putative range covered by this subspecies therefore extends across the interior of South Africa, and includes animals collected in the Western Cape (which group together with strong support; sourced from Visser, Bennett & Jansen van Vuuren (2019)) with an animal from Pretoria grouping basal and with lower support (sourced from Faulkes et al. (2004)). It is therefore possible that distinct genetic clades may exist within this subspecies. In general, intra-generic relationships were not consistently retrieved (with exception of the divergence between *C. h. natalensis* and *C. h. pretoriae*) and may indicate rapid and near contemporaneous divergences of groups. A degree of overlap exists in the ranges of *C. h. mahali* and *C. h. hottentotus* in the southern coastal parts of South Africa, as well as between *C. h. mahali* and *C. h. pretoriae* in the north-eastern interior of the country (Fig. 5B). Sequence divergence among the subspecies appear high and similar in values (8.1–10.3%; Fig. 5C; Table S7), with divergences largely contemporaneous during the Miocene (Fig. 5D).

#### Fukomys

**Figure 6:**
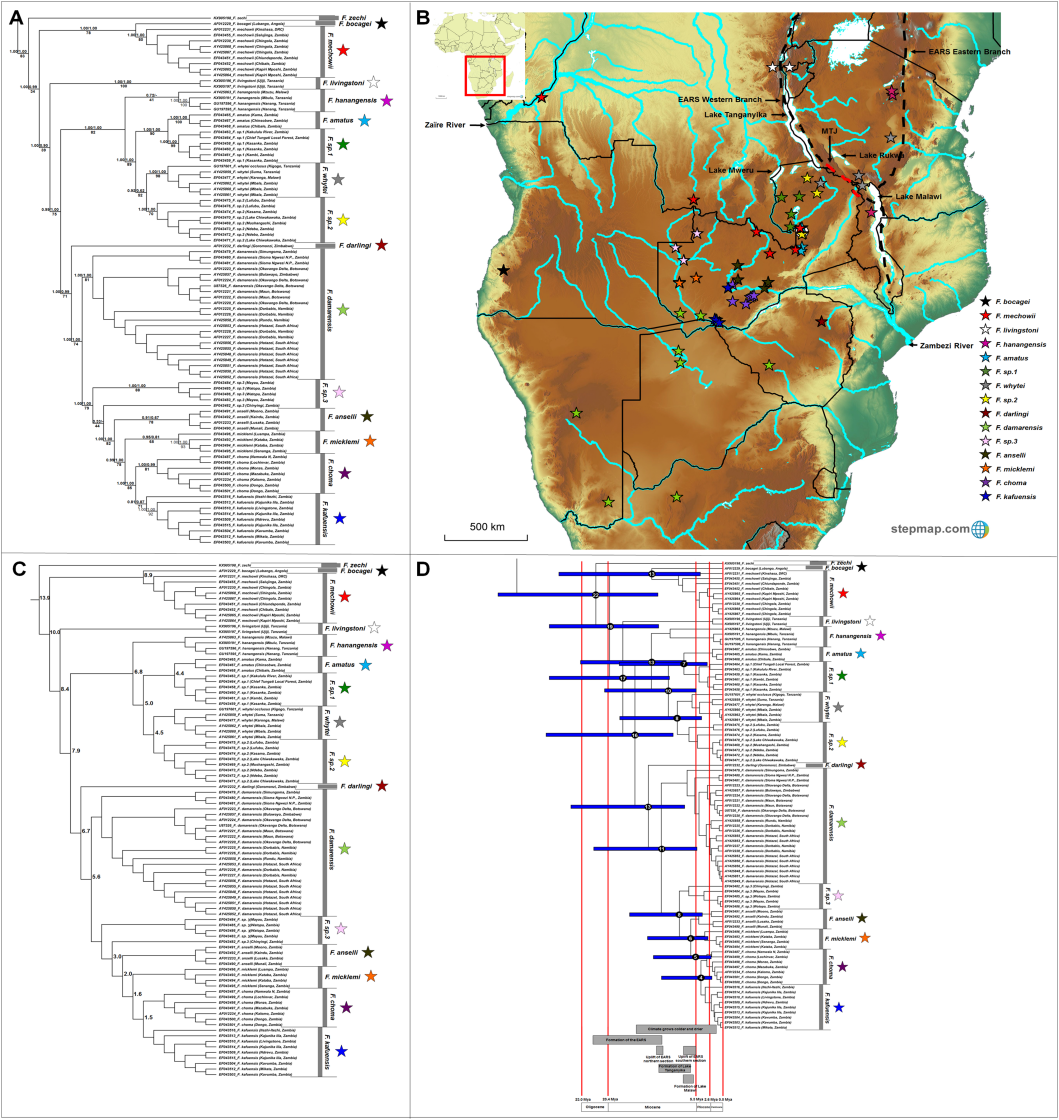
Phylogeny, geographic distributions, sequence divergences and divergence dates of species within the genus *Fukomys*. (A) Phylogeny of the genus *Fukomys* based on the cytochrome *b* data and indicating the different species included and identified. Values above nodes represent posterior probability values derived from the Bayesian analysis in BEAST and MrBayes respectively, while values below nodes indicate bootstrap values derived after Maximum Likelihood analysis in RAxML. A “-” indicates that the node was not retrieved by the particular analysis, while values in normal font show support for species which were recovered with the exclusion of a particular specimen. The geographic location (point locality for the specimen) of the recovered species is portrayed in (B) with the uncorrected sequence divergences (%) at the various nodes shown in (C). The divergence date estimates (in Mya) for each node is indicated within the black circles in (D), with the blue bars representing the temporal range of the divergence (derived from the dating analysis in BEAST). The red bars represent the temporal boundaries among the epochs, with the timing and temporal extent of major landscape changes which occurred across the distribution of the genus indicated at the bottom.

The genus *Fukomys* occupies a large range, with 16 major clades evident of which 12 are well-supported by all the analyses (Fig. 6A). Among the remaining clades, *Fukomys hanagensis*, *F. micklemi* and *F. kafuensis* also form well-supported monophyletic clades, when excluding sequences AY425863 (*F. hanagensis* from Mzuzu, Malawi), EF043496 (*F. micklemi* from Luampa, Zambia) and EF043516 (*F. kafuensis* from Itezhi-Ithezi, Zambia) from the analyses. The *F. anselli* clade received poor support irrespective method of analyses.

*Fukomys zechi* (the Ghana mole-rat) is the oldest extant lineage, followed by a number of species complexes and putatively new species distributed across Central, East and Southern Africa (Fig. 6A). Species distributions appear generally discrete, but a degree of overlap exists between the ranges of *F. mechowii*, *F. sp. 1* and *F. sp. 2* (Fig. 6B). Sequence divergences within *Fukomys* vary widely (1.5–16.2%; Fig. 6C; Table S8) resulting in a range of divergence times spanning the Oligocene to the more recent Plio/Pleistocene, but with the majority of events placed in the Miocene (Fig. 6D).

## Discussion

### Biogeographic patterns and species diversity

The Family Bathyergidae represent an interesting, unique, and highly successful group of sub-Saharan African subterranean mammals, with a range of unique adaptations to their fossorial lifestyle. Although much additional work needs to be done on the group to fully appreciate their evolution, it is clear that the Bathyergidae is characterized by a range of physiological adaptations, karyotype differences and genomic expressions (Deuve et al., 2008; Davies et al., 2015). Here, for the first time, we compile an inclusive phylogeny for the group, and attempt to unravel aspects of the group’s evolutionary and biogeographic histories. For this, we used the mitochondrial cytochrome *b* gene for which comparable data were available for all taxa. While the cytochrome *b* data were adequate at resolving intra-generic relationships and highlighting a number of putatively new species, deeper nodes within the phylogenetic tree (inter-generic divergences) remain less well resolved (but still in line with published data; see Allard & Honeycutt, 1992; Faulkes et al., 1997, 2004; Walton, Nedbal & Honeycutt, 2000; Ingram, Burda & Honeycutt, 2004; Deuve et al., 2008).

### Inter-generic patterns

The evolutionary placement of *Heterocephalus* outside the remaining five genera (*Heliophobius*, *Georychus*, *Bathyergus*, *Cryptomys* and *Fukomys*) offers strong support for the recognition of two subfamilies namely Heterocephalinae (for *Heterocephalus*) and Bathyerginae (for the remaining genera) (Ellerman, Morrison-Scott & Hayman, 1953). Within the Bathyerginae, *Heliophobiu*s is consistently retrieved as the oldest taxon (also see Allard & Honeycutt, 1992; Janecek et al., 1992; Faulkes et al., 1997, 2011; Walton, Nedbal & Honeycutt, 2000; Ingram, Burda & Honeycutt, 2004; Deuve et al., 2008 for a similar finding). The time of divergence of this genus from the other members of the subfamily is near-contemporaneous with the divergence of the two subfamilies (at approximately 35 Mya). These two oldest genera (*Heterocephalus* and *Heliophobius*) predominantly occur to the east of the Eastern Arc Rift System (see Fig. 1B).

Among the remaining genera, the sister relationship between the South African endemics *Georychus* and *Bathyergus* is consistently recovered (also see Faulkes et al., 1997, 2004; Walton, Nedbal & Honeycutt, 2000; Ingram, Burda & Honeycutt, 2004; Visser, Bennett & Jansen van Vuuren, 2019) and also the sister relationship between *Cryptomys* and *Fukomys* (also see Faulkes et al., 1997, 2004; Walton, Nedbal & Honeycutt, 2000; Ingram, Burda & Honeycutt, 2004). Although *Fukomys* largely occurs to the west of the Eastern Arc Rift System, some of its member-species straddle this barrier. A notable degree of range overlap exists between *Georychus* and *Bathyergus* in the south and south west of South Africa, although two *Georychus* species occur in the north-eastern parts of the country. *Cryptomys* is largely co-distributed with these genera across South Africa.

### Intra-generic patterns

The intra-generic taxonomy and evolutionary relationships within a number of mole-rat groups have always been contentious; largely as a result of the inconspicuous nature of the animals, and the difficulties of obtaining comprehensive samples across their ranges. For example, Ellerman (1940) proposed nine taxa in *Heliophobius*, but concluded that these constitute races of *Heliophobius argenteocinereus* rather than distinct species (with the exception of *Heliophobius spalax*). However, extensive karyotypic variation (George, 1979; Scharff, Macholan & Burda, 2001) and high levels of mitochondrial sequence divergences (Faulkes et al., 2004, 2011; Ingram, Burda & Honeycutt, 2004), led Faulkes et al. (2011) to propose species statuses for six geographic clades. We recover three distinct and well-supported genetic clades within this genus, although several specimens within each of these clades are highly divergent (e.g., JN244304 from the Nyika Plateau, as well as specimens from the Mbizi Mountains and Athi Plains) and may in fact represent putative species. Although the methods of analyses were not consistent in the relationships between these three clades, it would seem that the oldest species within *Heliophobius* (*H. sp. 2*) occurs to the east of the Eastern Arc Rift System (Fig. 2A). *Heliophobius sp. 1* is widespread across the Athi Plains and between the Eastern and Western Rifts in the Mbizi Mountains (Tanzania). Indications are that this may be a highland species, restricted to areas of higher elevation (Fig. 2B). In contrast, *Heliophobius argenteocinereus* occupies a coastal/lowland distribution across Tanzania. *Heliophobius sp. 2* occurs to the west of the Eastern Arc Rift System (although a single specimen was found east of the Eastern Arc Rift System in Mozambique), but similarly to *Heliophobius argenteocinereus*, follows an area of lower elevation along a north-south axis along, and crossing the Eastern Arc Rift System in an east-west axis in the southern part.

A single species is currently recognized for *Georychus* (*Georychus capensis*; type locality included in this study). However, our work suggests that there may be as many as four additionally undescribed species within *Georychus* (Fig. 3A). These unique genetic lineages geographically follow a clinal pattern: *G. sp. 1* and *G. sp. 2* are restricted to the north-eastern interior of South Africa and is found at higher elevations, *G. sp. 3* occurs in a single valley in the southern interior below the Great Escarpment, while *G*. *sp. 4* is located in the south-western coastal region (see Fig. 3B). *G. capensis* occurs further west, occupying a wide distribution in the interior (below the Great Escarpment) and lowland regions of south-western South Africa.

*Bathyergus* contains at least four species (Fig. 4A), two of which are the described species (*Bathyergus suillus* and *B*. *janetta*; type localities included in this study). All species are restricted to the southern and western seaboard of South Africa, with the exception of *B. janetta* which is found further north in the Namaqualand region and southern Namibia (Fig. 4B). *Bathyergus sp. 1* and *B. sp. 2* are restricted to the southern coastal margins and *B. suillus* to the western coastal margins.

Based on multi-locus genetic information, Visser, Bennett & Jansen van Vuuren (2019) recently proposed that the genus *Cryptomys* may be in need of a taxonomic revision, and suggested that *C. h. mahali*, *C. h. hottentotus* and *C. h. natalensis* should be elevated to species level. Considering all the currently described subspecies in this genus, the current study supports this (Fig. 5A, see also Faulkes et al., 2004; Ingram, Burda & Honeycutt, 2004). Although the relationships among the current subspecies within *Cryptomys* are not consistently retrieved (with the exception of the sister relationship between *C. h. natalensis* and *C. h. pretoriae*), the subspecies appear geographically exclusive. The sister clades *C. h. natalensis* and *C. h. pretoriae* are restricted to the eastern highland parts of South Africa (also see Ingram, Burda & Honeycutt, 2004), with *C. h. natalensis* occurring along the margins of the Great Escarpment and *C. h. pretoriae* further north-west in the Gauteng Province (see Fig. 5B). *C. h. hottentotus* occupies the coastal margins of south-western and north-western South Africa (also see Aguilar, 1993; Faulkes et al., 1997; Ingram, Burda & Honeycutt, 2004) with *C. h. nimrodi* found at higher elevations and the furthest north of all the current subspecies in Zimbabwe (also see Faulkes et al., 1997; Van Daele et al., 2004). *C. h. mahali* was collected in one area of high elevation around Pretoria (Faulkes et al., 1997, 2004), but also occurs in the low-lying coastal range of *C. h. hottentotus*. Although this would suggest a wide range for this group across South Africa, the genetic divergence between the Pretoria specimen and those from the coast suggest that these may be two highly divergent populations (possible subspecies). Additional sampling across the range is necessary to fully untangle the evolutionary relationships within the *mahali* group.

*Fukomys* includes a divergent and widespread collection of distinct genetic lineages (also see Faulkes et al., 1997, 2004, 2017; Ingram, Burda & Honeycutt, 2004; Van Daele et al., 2007b; Deuve et al., 2008) consisting of several species complexes of various evolutionary ages and with various degrees of divergence (Fig. 6; also see Van Daele et al., 2007b; Faulkes et al., 2017). The evolutionary progression within this groups suggests a West African origin, with subsequent migrations to the east and south (Fig. 6B). Specifically, *F. zechi* occupies the Sudanian savannah (Ghana) at the far north-western edge of the generic distribution, the sister species *F. bocagei* and *F. mechowii* are found to the far west (Angola) and along a northern band of the generic range respectively, and *F. livingstoni* occurs in the far north-east (in Tanzania, and east of the Eastern Arc Rift System). Similarly, *F. hanangensis* occurs in the far north-east of the distribution (in Tanzania and east of the Eastern Arc Rift System), albeit a single population is found west of the Eastern Arc Rift System (in Malawi). *F. darlingi* occupies the far south-east of the generic range in Zimbabwe.

Species of intermediate age (e.g., *F. amatus*, *F. sp. 1*, *F. whytei* and *F. sp. 2*) are located in a single geographic area to the west of the Eastern Arc Rift System (Fig. 6B), enclosed by Lakes Tanganyika, -Mweru Wantipa, -Mweru, -Bangweulu and -Malawi, as well as a single tributary of the Zaïre River system. Among these species, *F. whytei* occupies a range on both side of the Eastern Arc Rift System, with a distribution centered on and crossing the Mbeya Triple Junction, a volcanic area that formed 2 to 2.5 Mya (Ebinger et al., 1993; Delvaux et al., 1998; Macgregor, 2015) and forms an intersection between the two adjacent basins at the northern end of Lake Malawi (Fig. 6B; Faulkes et al., 2011). *F. damarensis* has a large distribution in the southern part of the generic distribution and to the south of the Zambezi River, while the most derived chromosomal races/species in *Fukomys* (*F. anselli*, *F. micklemi*, *F. choma*, *F. kafuensis* and the undescribed *F. sp. 3*) occur in south-central Zambia around tributaries of the Zambezi River (Fig. 6B).

### Major drivers of inter- and intra-generic divergences

Mole-rats are intimately linked to their subterranean niche, and it is not surprising that biogeographic patterns reviewed here suggest abrupt species turnover across various landscape features. It follows that evolutionary patterns within the Bathyergidae are heavily dependent on landscape structure, and the spatiotemporal variability thereof. Divergences among the genera, as well as among species within these genera, therefore coincide with prominent changes in landscape structure over evolutionary time.

The oldest inter-generic divergences probably coincide with early Miocene landscape changes. Climatically, warm and wet conditions prevailed during the early Miocene, which was interrupted by a progressively colder and drier phase during the middle Miocene (15.5–14 Mya, Siesser, 1980; Van Zinderen Bakker & Mercer, 1986; Zachos et al., 2001; Diekmann, Falker & Kuhn, 2003; Krammer, Baumann & Henrich, 2006). This favored the expansion of arid-adapted lineages (Axelrod & Raven, 1978; Coetzee, 1978, 1983; Bonnefille, 1984; Van Zinderen Bakker & Mercer, 1986), following a corridor of fluctuating aridity and a forest/woodland savannah mosaic across eastern, south-central and southern Africa (Van Zinderen Bakker, 1967; Van Couvering & Van Couvering, 1976; Kortlandt, 1983). Under these conditions ancestral bathyergids likely attained large distributions across sub-Saharan Africa, as supported by fossil bathyergids from the early Miocene deposits from East Africa and Namibia (Lavocat, 1973, 1978; also see Honeycutt et al., 1991; Faulkes et al., 2004). These wide distributions of the early bathyergids likely became fragmented through subsequent upheavals in the climate and geology of sub-Saharan Africa, driving spatial isolation and divergence within the group as a whole.

Major sculpting of the East African landscape began with the formation of the Eastern (Kenya) Rift at 23–11 Mya, with uplift of the Western Branch (Albertine Rift) of the Eastern Arc Rift System occurring at 12–11 Mya in the northern section and 7–5 Mya in the southern section (Van Couvering & Van Couvering, 1976; Ebinger, 1989; Ebinger et al., 1993). Tectonic activity continued into the Pliocene/Pleistocene (Ebinger, 1989; Ebinger et al., 1993; Bauer et al., 2010) and was instrumental in the formation of mountainous areas (e.g., the Eastern Arc Mountains at around 11–5.3 Mya) and volcanoes (see Chorowicz, 2005). This rifting process also drove the formation of the Great Lakes (Lake Tanganyika 12–6 Mya, Lake Malawi 7.2–5.3 Mya, Cohen, Soreghan & Scholz, 1993; Delvaux, 1995; Delvaux et al., 1998; Macgregor, 2015).

The early divergence of the East African *Heterocephalus* and *Heliophobius* appears independent of rifting (also see Faulkes et al., 2017), while the divergence between the two generic complexes of *Georychus*/*Bathyergus* and *Cryptomys*/*Fukomys* may have followed the spatial separation of a common ancestor via different routes of dispersal from East Africa. Within the *Georychus*/*Bathyergus* complex, a widespread common ancestor likely diverged parapatrically, with *Georychus* evolving in the South African interior and *Bathyergus* diverging within sandy areas of south and south-western South Africa. Divergence between *Cryptomys* and *Fukomys* follows the Zambezian and the Kalahari-Highveld phytochoria and was likely driven by the pattern of flow of the paleo-Zambezi River (the upper Zambezi River was linked to the Orange- or Limpopo Rivers; Thomas & Shaw, 1988). This flow pattern isolated ancestors of the two genera, with *Cryptomys* radiating southward while *Fukomys* spread north and west to attain a large distribution in south-central and West Africa (also see Ingram, Burda & Honeycutt, 2004; Faulkes et al., 2017).

The major historical landscape changes across East Africa coincide with intra-generic speciation patterns within the genera *Heliophobius* and *Fukomys* (Figs. 2D and 6D). The formation of the Eastern Arc Rift System and the Great Lakes likely fragmented ancestral *Heliophobius* populations, subsequently leading to the divergence of *H. sp. 1* and *H. sp. 2*/*Heliophobius argenteocinereus* across this newly formed barrier. Elevation differences created through uplift (e.g., of the Eastern Arc Mountains) across this tectonically active region also likely restricted individual *Heliophobius* species to either highland (*H. sp. 1*) or lowland (*H. sp. 2*/*Heliophobius argenteocinereus*) habitats. Formation of Lakes Rukwa and Malawi also appear instrumental in the divergence between *F. whytei* and *F. sp. 2*, from where *F. whytei* subsequently expanded its range eastward via the Mbeya Triple Junction following this divergence (*F. whytei* lineages follow a geographically clinal pattern in an eastward direction; Fig. 6A). In contrast, *F. hanangensis* appears to have originated on the western side of the Eastern Arc Rift System, and has subsequently colonized the eastern side in Tanzania, also likely following the Mbeya Triple Junction (the oldest *F. hanangensis* lineage is found west of the Eastern Arc Rift System; Fig. 6A).

Aside from impact of rifting in driving divergence among the above *Fukomys* species, the wider distribution of this genus has been subjected to the consequences of uplift across central and eastern Africa. This undoubtedly influenced drainage evolution in the paleo-Zambezi and paleo-Zaïre River watersheds. Initial drainage evolution of the paleo-Zaïre River watershed (specifically the paleo-Kafue River and Luapula River), along with the formation of lakes (Lakes Tanganyika, -Mweru Wantipa, -Mweru, -Bangweulu and -Malawi), appear to have driven speciation within the *F. hanangensis*, *F. amatus*, *F. sp. 1*, *F. sp. 2* species complex. This pattern of isolation is also mirrored in extra-limital species of a similar evolutionary age (*F. darlingi* and *F. damarensis*) around the Zambezi River watershed. Continued drainage evolution of the Zambezi River watershed across central Zambia along with geomorphological repatterning of the area, appear to have driven speciation among the younger *Fukomys* species (e.g., *F. sp. 3*, *F. anselli*, *F. micklemi*, *F. choma* and *F. kafuensis*) which are separated by branches of the Zambezi River system. Furthermore, these species exhibit extensive chromosomal rearrangements, possibly following ecological specialization to separate areas in this ecogeographically heterogeneous region (Van Daele et al., 2007b).

Aside from the East African genera, speciation within the three southern African genera *Georychus*, *Bathyergus* and *Cryptomys* exclusively follow geological and climatic changes across this sub-region. In South Africa, early Miocene uplift initiated the Post-African I erosion cycle (Partridge & Maud, 1987) with a second and more pronounced uplift event in the late Miocene/early Pliocene leading to the Post-African II erosion cycle (Partridge & Maud, 1987, 2000). Cold and dry episodes dominated the climate since the middle Miocene (Van Zinderen Bakker & Mercer, 1986) and late Miocene (Shackleton & Kennett, 1975a, 1975b; Van Zinderen Bakker & Mercer, 1986; Tyson & Partridge, 2000), with a cold period at the Miocene/Pliocene boundary (5 Mya) leading to the abolishment of subtropical rainforests in south-western South Africa (Van Zinderen Bakker & Mercer, 1986). Following glacial cycles, oceanic fluctuations exposed or inundated large areas of the coastal shelf (more than 500 km) during oceanic regression and transgression phases respectively (Dingle & Rogers, 1972; Siesser & Dingle, 1981; Maud & Partridge, 1987; Rogers, 1987; Hallam, 1992). A major regression lasted from the early Oligocene up until the end of the early Miocene (low-stand of more than 500 m below present during the Oligocene/Miocene boundary), while a major regression occurred from the middle Miocene lasting until the Pliocene (high-stand of up to 300 m above present during the late Miocene/early Pliocene; Siesser & Dingle, 1981).

Radiations within *Georychus*, *Bathyergus* and *Cryptomys* followed the Post-African I erosion cycle. Prior to this uplift event, the topography across southern Africa would have been more uniform. During this period, a major sea-level regression was also underway (Dingle & Rogers, 1972; Siesser & Dingle, 1981). Under these condition, ancestral lineages in all three genera were probably widespread across the interior as well as along the coastal margins of southern Africa. These wide distributions would have been bisected by the first major uplift event which initiated the Post-African I erosion cycle. This uplift raised the interior plateau of southern Africa (250–300 m in the east and 150–200 m in the west), creating a rugged and sloping topography as well as mountainous areas across the sub-region, consequently leading to the incision of deep river valleys (Partridge & Maud, 1987; Cowling, Procheş & Partridge, 2009).

As a result, divergences in the largely co-distributed genera *Georychus* and *Bathyergus* (and possibly *Cryptomys*) follow elevation differences, mountain barriers and drainage evolution of the major river systems. The influence of elevation differences is evident in the occurrence of several highland species which occupy the southern African interior (e.g., *G. sp. 1*, *G. sp. 2*, *C. h. pretoriae*, *C. h. natalensis* and possibly *C. h. mahali*), in contrast to several lowland/coastal species (e.g., *G. sp. 3*, *G. sp. 4*, *G. capensis* and *C. h. hottentotus*). Mountainous areas such as the Hottentots Holland Mountains also forms a major geographic barrier between *B. suillus* and *B. sp. 1*/*B. sp. 2*. Similar to *Fukomys*, the influence of drainage evolution as an isolating factor is apparent, with the Breede and Gouritz rivers forming major geographic barriers between *B. sp. 1* and *B. sp. 2*, and *G. sp. 3*, *G. sp. 4* and *G. capensis*, and drainage evolution of the Limpopo River likely leading to the divergence of *C. h. nimrodi*. Given that a large part of the distributions of *Georychus* and *Bathyergus* are exclusively coastal, the major sea-level transgression which covered the coastal shelf during the late Miocene/early Pliocene also appears instrumental in driving/enforcing isolation of the lowland species such as *B. sp. 1* and *B. sp. 2*, *B. suillus* and *B. janetta*, and *G. sp. 4*.

### Possible other factors driving speciation within the Bathyergidae

Although physical landscape changes were undoubtedly instrumental in driving radiations within the Bathyergidae, these events alone cannot explain all the divergences within the family. Historical climatic changes, intrinsic population-level responses or ecological specialization of species undoubtedly contributed (Faulkes et al., 2004, 2010; Van Daele et al., 2007b; Visser, Bennett & Jansen van Vuuren, 2019) although these drivers are frequently overlooked in phylogeoraphic/systematic studies on the Bathyergidae (but see Van Daele et al., 2007b).

Given the ecogeographical turnover across the African landscape, isolation in fringe habitat indicates the possibility that geographically distant species represent narrow endemics which are adapted to localized and diminishing habitat types. In contrast, the majority of younger or less divergent species occupy a central distribution, where suitable habitat (similar ecological circumstances) is likely abundant and divergences largely follow vicariant events. Understanding and incorporating species-specific responses/attributes (e.g., ecological and demographic information) may therefore be critical during phylogeographic/systematic endeavors in the Bathyergidae—a paradigm shift which is becoming increasingly important when interpreting genetic patterns across landscapes (Papadopouloua & Knowles, 2016).

Indeed, the geographic ranges of a large number of mole-rat lineages are spatially limited, and secondary contact is uncommon even in areas without apparent physical barriers. Given the heterogeneous geology, microclimate and phytogeography of Africa across various spatial scales, it is reasonable to assume that ecological and/or adaptive differences exist among species across their respective ranges, and that other intrinsic and extrinsic processes may be of importance in driving or enforcing isolation. As such, the Bathyergidae may include a multitude of habitat specialist taxa. These specialists may be prone to extinction through stochastic population processes, or modern perturbations which diminish preferred habitat (e.g., current levels of climate change).

Highly divergent and geographically restricted species within three of the genera is found at the edges of their respective generic ranges. No obvious link to physical landscape evolution explains the origin or distribution of the oldest species within *Fukomys* (e.g., *F. zechi*, *F. bocagei*, *F. livingstoni*). These species are separated from other members of the genus by large geographic distances, and likely occupy diminishing fringe habitats in the generic range. Similarly, large distances separate species within the genus *Georychus*. It is entirely possible that these (or at least *G. sp. 1* and *2*) species represent relic populations, isolated in pockets of suitable habitat. Similarly, *F. zechi* was likely isolated form congenerics by the formation of the tropical rainforest in the Congo Basin (Ingram, Burda & Honeycutt, 2004; Van Daele et al., 2007a; Faulkes et al., 2017).

### Bathyergid systematics—historical trends and future directions

The data presented here confirms the notion that allopatric speciation is prevalent in taxa which inhabit the fossorial/subterranean niche (Fitzpatrick & Turelli, 2006). Phylogeographic/spatial genetic studies have consistently recovered patterns of intimate co-evolution between fossorial taxa and the landscape, invariably finding genetically discrete lineages or species in separate geographic regions (Albert, Zardoya & García-París, 2007; Opazo et al., 2008; Tang et al., 2010).

While the patterns recovered within fossorial taxa have remained broadly similar, the choice of markers for inferring genetic isolation has grown by leaps and bounds over the past four decades. Initially, inferences of genetically discrete groups were based on karyotypic and allozyme information (Nevo et al., 1974; Patton & Yang, 1977; Hafner et al., 1987). With the advent of more refined genetic sequencing techniques, mitochondrial DNA sequence data dominated fossorial phylogeography and systematics (Albert, Zardoya & García-París, 2007; Opazo et al., 2008; Tang et al., 2010) while more recently, the addition of nuclear markers has aided in the discovery of cryptic species and discrete lineages (Bannikova et al., 2015; Salvi et al., 2018).

Aside from these developments in the types and amount of data generated in systematic studies, there have also been developments in linking genetic patterns (especially adaptive genetic patterns) with the ecogeographic characteristics of the landscape. Adaptive differences (ecological, physiological, behavioral, morphological and genetic) in the fossorial genus *Spalax* have been intensively studied over the past three decades in four parapatric chromosomal species distributed across an ecogeographical and climatic gradient in Israel (Nevo & Bar-El, 1976; Nevo, 1985; Nevo et al., 1994, 2000). To some extent, similar patterns have been demonstrated in the fossorial genera *Nanospalax* (Savić, Ćirović & Bugarski-Stanojević, 2017) and *Geomys* (Heaney & Timm, 1985). Based on advances in genomic sequencing abilities, more recent studies have successfully employed data on adaptive genes and gene expression (whole mitochondrial and nuclear DNA genomes, transcriptomes, DNA methylation, micro RNA and codon usage) to show adaptive ecological speciation between two *Spalax* species which inhabit adjacent areas contrasting sharply in geology, edaphic attributes, vegetation and ecology (Hadid et al., 2012; Lövy et al., 2015; Li et al., 2015, 2016; Zhao et al., 2016). In these studies, it was shown that natural selection has acted on >300 genes to create adaptive complexes in these sharply contrasting ecogeographic areas.

By comparison, systematic revisions within the Bathyergidae have kept pace with the development of approaches. These revisions were, however, not consistent in the use of specific data types with some studies based on karyotype (Aguilar, 1993; Kawalika, Burda & Brüggert, 2001; Van Daele et al., 2004; Burda et al., 2005; Deuve et al., 2008) or allozyme data (Janecek et al., 1992; Filipucci et al., 1997) or mitochondrial DNA sequence data (Allard & Honeycutt, 1992; Faulkes et al., 1997, 2004, 2010, 2011, 2017; Van Daele et al., 2007b). Only in single instances (and more recently) have information from nuclear markers been included (Walton, Nedbal & Honeycutt, 2000; Huchon & Douzery, 2001; Ingram, Burda & Honeycutt, 2004; Kundu & Faulkes, 2004; Visser, Bennett & Jansen van Vuuren, 2014, 2018, 2019).

A significant advance in bathyergid systematics entailed the investigation of adaptive genes and gene complexes among the six bathyergid genera (Davies et al., 2015). Here, more than 700 genes were identified which convey some form of adaptive function. Based upon this information, Visser, Bennett & Jansen van Vuuren (2019) selected three genetic markers (in addition to mitochondrial DNA sequence data) to investigate genetic exclusiveness across the distributions of the two South African bathyergid genera *Bathyergus* and *Georychus*. The authors also demonstrated differences in the ecology (and to some extent the mating system in *Georyhcus*) among the recovered genetically and geographically discrete groups. More importantly, the discrete groups were recovered using both the adaptive nuclear data, as well as the cytochrome *b* data, indicating that this marker may be adequate for exploring the existence of highly divergent lineages.

From the cytochrome *b* and distributional data reviewed here, the possibility is highlighted that several cryptic and yet undescribed taxa exist within the Bathyergidae. In addition, these and many other bathyergid taxa may represent narrow endemics, frequently occupying diminishing habitats. An urgent and thorough systematic revision of the family may therefore be a necessity, especially given the rate of extinction linked to environmental change (Ceballos et al., 2015; Ocampo-Peñuela et al., 2016). The phylogeny presented here may serve as an adequate starting point for future taxonomic endeavors. Readers who have access to the material used in this study are urged to sequence additional and variable or adaptive nuclear markers to enable formal species descriptions. Additional sampling may also be required across wider geographic ranges and including larger sample sizes and more localities, so as to define species distributions- and relationships. A thorough systematic investigation of the Bathyergidae should also aim to include ecological and/or demographic data when defining species integrities. As such, this may aid in the identification of narrow endemics, as well as in conservation initiatives which aim to conserve the suitable habitat which harbors these species.

## Conclusions

Here, we presented for the first time a comprehensive phylogeny for the Bathyergidae based on inclusive sampling across the ranges of genera and species, and place our phylogeny within the context of the species’ biologies and Africa’s complex geological and climatic history. We confirm the East African origin for the Bathyergidae (Faulkes et al., 2004, 2011, 2017) but suggest that earlier radiations were independent of rifting. Biogeographic patterns of the genera bear a signature of wide historical distributions (likely following open and savannah type habitats) which were fragmented by Miocene landscape changes. Importantly, the effects of climatic changes, distribution of suitable habitat, habitat specialization and population-level processes remain largely unexplored, but may have acted either unattended or in concert with vicariant events to influence speciation patterns.

Although largely exploratory in nature, it appears that several undescribed species may exist within the Bathyergidae, with five of the six genera harboring multiple species and possibly even undescribed cryptic species—many of which may be narrow endemics. As such, three species are recovered in *Heliophobius* (*Heliophobius argenteocinereus*, *H. sp. 1* and *H. sp. 2*), five species in *Georychus* (*G. capensis*, *G. sp. 1*, *G. sp. 2*, *G. sp. 3* and *G. sp. 4*), four species in *Bathyergus* (*B. suillus*, *B. janetta*, *B. sp. 1* and *B. sp. 2*), five species in *Cryptomys* (*C. h. mahali*, *C. h. nimrodi*, *C. h. hottentotus*, *C. h. natalensis* and *C. h. preoriae*) and 16 species in *Fukomys* (*F. zechi*, *F. bocagei*, *F. mechowii*, *F. livingstoni*, *F. hanangensis*, *F. amatus*, *F. whytei*, *F. darlingi*, *F. damarensis, F. anselli*, *F. micklemi*, *F. choma*, *F. kafuensis*, and *F. sp. 1*, *F. sp. 2* and *F. sp. 3*). Given the current rate of loss in biodiversity worldwide, a thorough systematic review of the Bathyergidae is crucial to discover and conserve the patterns and drivers of biodiversity in the family. Such a review would require intensive sampling, large sample sizes, multiple genetic markers and the addition of ecological and/or demographic data.

## Supplemental Information

10.7717/peerj.7730/supp-1 Supplemental Information 1 Phylogeny of the Bathyergidae based on cytochrome *b* sequence data. Phylogeny of the Bathyergidae constructed through Bayesian analysis (in BEAST) and based on the cytochrome *b* sequence data for all the available sample haplotypes listed in Table S1. The various mole-rat species included and identified within each genus are indicated. Values above nodes represent posterior probability values derived from the Bayesian analysis in BEAST and MrBayes respectively, while values below nodes indicate bootstrap values derived after Maximum Likelihood analysis in RAxML.

10.7717/peerj.7730/supp-2 Supplemental Information 2 Specimen/sequence information of the bathyergids included in this study. Accession numbers of the cytochrome *b* haplotypes used in this study, also indicating the species (as designated on the public database), reference to the original study in which the sequence was generated, the species designation in the original study, the species designation in the current study and geographic information and coordinates of the sampling locality from where the species originates.

10.7717/peerj.7730/supp-3 Supplemental Information 3 Species authorities on the bathyergid species included in this study.

10.7717/peerj.7730/supp-4 Supplemental Information 4 Uncorrected sequence divergences among the six bathyergid genera. Pairwise estimates of uncorrected sequence divergence among the six bathyergid genera.

10.7717/peerj.7730/supp-5 Supplemental Information 5 Uncorrected sequence divergences among the species within *Heliophobius*. Pairwise estimates of uncorrected sequence divergence among the various species included and identified within the genus *Heliophobius*.

10.7717/peerj.7730/supp-6 Supplemental Information 6 Uncorrected sequence divergences among the species within *Georychus*. Pairwise estimates of uncorrected sequence divergence among the various species included and identified within the genus *Georychus*.

10.7717/peerj.7730/supp-7 Supplemental Information 7 Uncorrected sequence divergences among the species within *Bathyergus*. Pairwise estimates of uncorrected sequence divergence among the various species included and identified within the genus *Bathyergus*.

10.7717/peerj.7730/supp-8 Supplemental Information 8 Uncorrected sequence divergences among the species within *Cryptomys*. Pairwise estimates of uncorrected sequence divergence among the various species (subspecies) included and identified within the genus *Cryptomys*.

10.7717/peerj.7730/supp-9 Supplemental Information 9 Uncorrected sequence divergences among the species within *Fukomys*. Pairwise estimates of uncorrected sequence divergence among the various species included and identified within the genus *Fukomys*.

## Additional Information and Declarations

### Data Availability

The following information was supplied regarding data availability: The data used in this study are available from public databases (EMBL and Genbank). Their accession numbers are available in the Supplemental File.
