# Supplementary figures and images for: Phylogeny and biogeography of the African Bathyergidae: a review of patterns and processes
Source: PeerJ. 2019 Oct 15;7:e7730. doi: 10.7717/peerj.7730 (PMC6798870; doi:10.7717/peerj.7730)

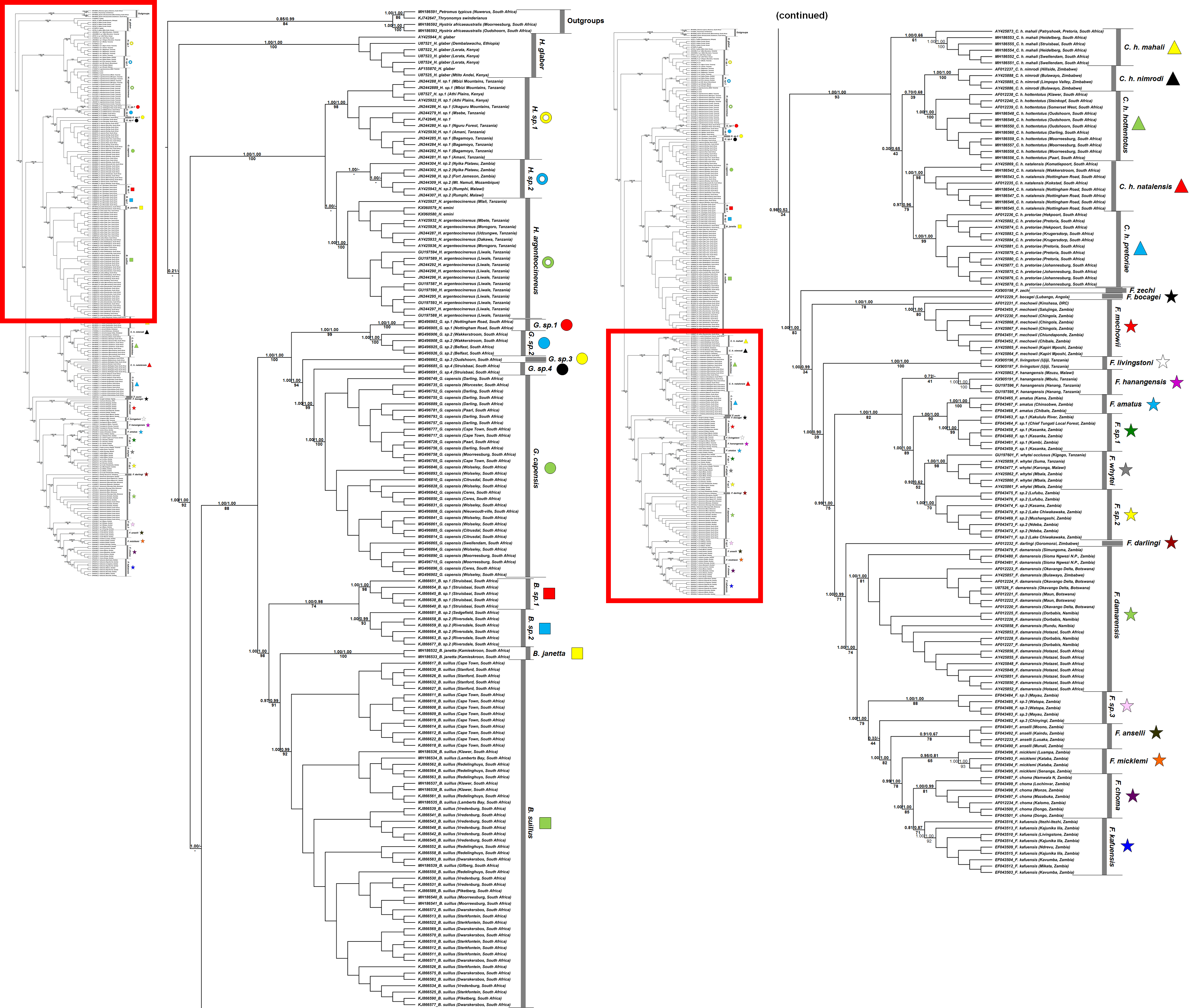

Supplement: Supplemental Information 1 — Phylogeny of the Bathyergidae constructed through Bayesian analysis (in BEAST) and based on the cytochrome b sequence data for all the available sample haplotypes listed in Table S1. The various mole-rat species included and identified within each genus are indicated. Values above nodes represent posterior probability values derived from the Bayesian analysis in BEAST and MrBayes respectively, while values below nodes indicate bootstrap values derived after Maximum Likelihood analysis in RAxML. [file peerj-07-7730-s001.png]
