# Supplementary material for: Phylogeny and biogeography of the African Bathyergidae: a review of patterns and processes
Source: PeerJ. 2019 Oct 15;7:e7730. doi: 10.7717/peerj.7730 (PMC6798870; doi:10.7717/peerj.7730)
Supplement: Supplemental Information 2 — Accession numbers of the cytochrome b haplotypes used in this study, also indicating the species (as designated on the public database), reference to the original study in which the sequence was generated, the species designation in the original study, the species designation in the current study and geographic information and coordinates of the sampling locality from where the species originates. [file peerj-07-7730-s002.docx]

| ***Heterocephalus*** | | | | | | |
| --- | --- | --- | --- | --- | --- | --- |
| **Accession number** | **Species designation: Public database** | **Original study** | **Species designation: Original study** | **Species designation: This study** | **Geographic information** | **Coordinates** |
| AF155870 | *Heterocephalus glaber* | - | *-* | *Heterocephalus glaber* | - | - |
| AY425944 | *Heterocephalus glaber* | - | *-* | *Heterocephalus glaber* | - | - |
| U87521 | *Heterocephalus glaber* | *Faulkes et al., 2004* | *Heterocephalus glaber* | *Heterocephalus glaber* | Dembalawachu, Ethiopia | N 04°53'; E 38°06' |
| U87522 | *Heterocephalus glaber* | *Faulkes et al., 2004* | *Heterocephalus glaber* | *Heterocephalus glaber* | Lerata, Kenya | N 00°37'; E 37°39' |
| U87523 | *Heterocephalus glaber* | *Faulkes et al., 2004* | *Heterocephalus glaber* | *Heterocephalus glaber* | Lerata, Kenya | N 00°37'; E 37°39' |
| U87524 | *Heterocephalus glaber* | *Faulkes et al., 2004* | *Heterocephalus glaber* | *Heterocephalus glaber* | Lerata, Kenya | N 00°37'; E 37°39' |
| U87525 | *Heterocephalus glaber* | *Faulkes et al., 2004* | *Heterocephalus glaber* | *Heterocephalus glaber* | Mtito Andei, Kenya | S 02°41'; E 38°09' |
| ***Heliophobius*** | | | | | | |
| **Accession number** | **Species designation: Public database** | **Original study** | **Species designation: Original study** | **Species designation: This study** | **Geographic information** | **Coordinates** |
| U87527 | *Heliophobius argenteocinereus* | *Faulkes et al., 2011* | *Heliophobius sp.* | *Heliophobius Sp.1* | Athi Plains, Kenya | S 01°43'; E 37°00' |
| AY425922 | *Heliophobius argenteocinereus* | *Faulkes et al., 2011* | *Heliophobius sp.* | *Heliophobius Sp.1* | Athi Plains, Kenya | S 01°43'; E 37°00' |
| JN244288 | *Heliophobius sp.* | *Faulkes et al., 2011* | *Heliophobius sp.* | *Heliophobius Sp.1* | Mbizi Mountains, Tanzania | S 07°37'; E 31°08' |
| JN244289 | *Heliophobius sp.* | *Faulkes et al., 2011* | *Heliophobius sp.* | *Heliophobius Sp.1* | Mbizi Mountains, Tanzania | S 07°37'; E 31°08' |
| JN244286 | *Heliophobius sp.* | *Faulkes et al., 2011* | *Heliophobius sp.* | *Heliophobius Sp.1* | Ukaguru Mountains, Tanzania | S 06°28'; E 36°50' |
| AY425930 | *Heliophobius argenteocinereus* | *Faulkes et al., 2011* | *Heliophobius sp.* | *Heliophobius Sp.1* | Amani, Tanzania | S 04°59'; E 38°39' |
| JN244281 | *Heliophobius sp.* | *Faulkes et al., 2011* | *Heliophobius sp.* | *Heliophobius Sp.1* | Amani, Tanzania | S 04°59'; E 38°39' |
| JN244282 | *Heliophobius sp.* | *Faulkes et al., 2011* | *Heliophobius sp.* | *Heliophobius Sp.1* | Bagamoyo, Tanzania | S 05°06'; E 38°24' |
| JN244284 | *Heliophobius sp.* | *Faulkes et al., 2011* | *Heliophobius sp.* | *Heliophobius Sp.1* | Bagamoyo, Tanzania | S 05°06'; E 38°24' |
| JN244285 | *Heliophobius sp.* | *Faulkes et al., 2011* | *Heliophobius sp.* | *Heliophobius Sp.1* | Bagamoyo, Tanzania | S 05°06'; E 38°24' |
| KJ742646 | *Heliophobius argenteocinereus* | - | - | *Heliophobius Sp.1* | - | - |
| JN244279 | *Heliophobius sp.* | *Faulkes et al., 2011* | *Heliophobius sp.* | *Heliophobius Sp.1* | Msebe, Tanzania | S 06°43'; E 37°37' |
| JN244280 | *Heliophobius sp.* | *Faulkes et al., 2011* | *Heliophobius sp.* | *Heliophobius Sp.1* | Nguru Forest, Tanzania | S 06°39'; E 37°35' |
| JN244304 | *Heliophobius sp.* | *Faulkes et al., 2011* | *Heliophobius sp.* | *Heliophobius Sp.2* | Nyika Plataeu, Zambia | S 10°28'; E 33°30' |
| AY425943 | *Heliophobius argenteocinereus* | *Faulkes et al., 2011* | *Heliophobius sp.* | *Heliophobius Sp.2* | Rumphi, Malawi | S 11°01'; E 33°52' |
| JN244307 | *Heliophobius sp.* | *Faulkes et al., 2011* | *Heliophobius sp.* | *Heliophobius Sp.2* | Rumphi, Malawi | S 11°01'; E 33°52' |
| JN244309 | *Heliophobius sp.* | *Faulkes et al., 2011* | *Heliophobius sp.* | *Heliophobius Sp.2* | Mt. Namuli, Mozambique | S 15°26'; E 37°03' |
| JN244298 | *Heliophobius sp.* | *Faulkes et al., 2011* | *Heliophobius sp.* | *Heliophobius Sp.2* | Fort Jameson, Zambia | S 13°37'; E 32°38' |
| JN244302 | *Heliophobius sp.* | *Faulkes et al., 2011* | *Heliophobius sp.* | *Heliophobius Sp.2* | Nyika Plataeu, Zambia | S 10°28'; E 33°30' |
| AY425926 | *Heliophobius argenteocinereus* | *Faulkes et al., 2011* | *Heliophobius sp.* | *Heliophobius argenteocinereus* | Morogoro, Tanzania | S 06°50'; E 37°39' |
| AY425932 | *Heliophobius argenteocinereus* | *Faulkes et al., 2011* | *Heliophobius sp.* | *Heliophobius argenteocinereus* | Mbete, Tanzania | S 06°51'; E 37°40' |
| AY425933 | *Heliophobius argenteocinereus* | *Faulkes et al., 2011* | *Heliophobius sp.* | *Heliophobius argenteocinereus* | Dakawa, Tanzania | S 06°26'; E 37°34' |
| AY425936 | *Heliophobius argenteocinereus* | *Faulkes et al., 2011* | *Heliophobius sp.* | *Heliophobius argenteocinereus* | Morogoro, Tanzania | S 06°50'; E 37°39' |
| JN244287 | *Heliophobius sp.* | *Faulkes et al., 2011* | *Heliophobius sp.* | *Heliophobius argenteocinereus* | Udzungwe, Tanzania | S 08°09'; E 36°13' |
| KX060579 | *Heliophobius emini* | - | - | *Heliophobius emini* | - | - |
| KX060580 | *Heliophobius emini* | - | - | *Heliophobius emini* | - | - |
| AY425927 | *Heliophobius argenteocinereus* | *Faulkes et al., 2011* | *Heliophobius sp.* | *Heliophobius argenteocinereus* | Mlali, Tanzania | S 06°57'; E 37°40' |
| JN244290 | *Heliophobius sp.* | *Faulkes et al., 2011* | *Heliophobius sp.* | *Heliophobius argenteocinereus* | Liwale, Tanzania | S 09°46'; E 37°57' |
| JN244292 | *Heliophobius sp.* | *Faulkes et al., 2011* | *Heliophobius sp.* | *Heliophobius argenteocinereus* | Liwale, Tanzania | S 09°46'; E 37°57' |
| JN244295 | *Heliophobius sp.* | *Faulkes et al., 2011* | *Heliophobius sp.* | *Heliophobius argenteocinereus* | Liwale, Tanzania | S 09°46'; E 37°57' |
| JN244296 | *Heliophobius sp.* | *Faulkes et al., 2011* | *Heliophobius sp.* | *Heliophobius argenteocinereus* | Liwale, Tanzania | S 09°46'; E 37°57' |
| JN244297 | *Heliophobius sp.* | *Faulkes et al., 2011* | *Heliophobius sp.* | *Heliophobius argenteocinereus* | Liwale, Tanzania | S 09°46'; E 37°57' |
| GU197587 | *Heliophobius sp.* | *Faulkes et al., 2011* | *Heliophobius sp.* | *Heliophobius argenteocinereus* | Liwale, Tanzania | S 09°46'; E 37°57' |
| GU197588 | *Heliophobius sp.* | *Faulkes et al., 2010* | *Heliophobius sp.* | *Heliophobius argenteocinereus* | Liwale, Tanzania | S 09°46'; E 37°57' |
| GU197589 | *Heliophobius sp.* | *Faulkes et al., 2010* | *Heliophobius sp.* | *Heliophobius argenteocinereus* | Liwale, Tanzania | S 09°46'; E 37°57' |
| GU197590 | *Heliophobius sp.* | *Faulkes et al., 2010* | *Heliophobius sp.* | *Heliophobius argenteocinereus* | Liwale, Tanzania | S 09°46'; E 37°57' |
| GU197593 | *Heliophobius sp.* | *Faulkes et al., 2010* | *Heliophobius sp.* | *Heliophobius argenteocinereus* | Liwale, Tanzania | S 09°46'; E 37°57' |
| GU197594 | *Heliophobius sp.* | *Faulkes et al., 2010* | *Heliophobius sp.* | *Heliophobius argenteocinereus* | Liwale, Tanzania | S 09°46'; E 37°57' |
| ***Georychus*** | | | | | | |
| **Accession number** | **Species designation: Public database** | **Original study** | **Species designation: Original study** | **Species designation: This study** | **Geographic information** | **Coordinates** |
| MG496903 | *Georychus capensis* | *Visser, Bennett & Jansen van Vuuren, 2018* | *Georychus capensis* | *Georychus sp.1* | Nottingham Road, South Africa | S 29°29'; E 29°52' |
| MG496905 | *Georychus capensis* | *Visser, Bennett & Jansen van Vuuren, 2018* | *Georychus capensis* | *Georychus sp.1* | Nottingham Road, South Africa | S 29°29'; E 29°52' |
| MG496906 | *Georychus capensis* | *Visser, Bennett & Jansen van Vuuren, 2018* | *Georychus capensis* | *Georychus sp.2* | Wakkerstroom, South Africa | S 27°18'; E 30°16' |
| MG496908 | *Georychus capensis* | *Visser, Bennett & Jansen van Vuuren, 2018* | *Georychus capensis* | *Georychus sp.2* | Wakkerstroom, South Africa | S 27°18'; E 30°16' |
| MG496925 | *Georychus capensis* | *Visser, Bennett & Jansen van Vuuren, 2018* | *Georychus capensis* | *Georychus sp.2* | Belfast, South Africa | S 25°33'; E 30°04' |
| MG496926 | *Georychus capensis* | *Visser, Bennett & Jansen van Vuuren, 2018* | *Georychus capensis* | *Georychus sp.2* | Belfast, South Africa | S 25°33'; E 30°04' |
| MG496663 | *Georychus capensis* | *Visser, Bennett & Jansen van Vuuren, 2018* | *Georychus capensis* | *Georychus sp.3* | Oudshoorn, South Africa | S 33°51'; E 22°02' |
| MG496685 | *Georychus capensis* | *Visser, Bennett & Jansen van Vuuren, 2018* | *Georychus capensis* | *Georychus sp.4* | Struisbaai, South Africa | S 34°41'; E 20°00' |
| MG496691 | *Georychus capensis* | *Visser, Bennett & Jansen van Vuuren, 2018* | *Georychus capensis* | *Georychus sp.4* | Struisbaai, South Africa | S 34°41'; E 20°00' |
| MG496808 | *Georychus capensis* | *Visser, Bennett & Jansen van Vuuren, 2018* | *Georychus capensis* | *Georychus capensis* | Nieuwoudt-ville, South Africa | S 31°22'; E 19°06' |
| MG496814 | *Georychus capensis* | *Visser, Bennett & Jansen van Vuuren, 2018* | *Georychus capensis* | *Georychus capensis* | Citrusdal, South Africa | S 32°36'; E 19°01' |
| MG496885 | *Georychus capensis* | *Visser, Bennett & Jansen van Vuuren, 2018* | *Georychus capensis* | *Georychus capensis* | Citrusdal, South Africa | S 32°36'; E 19°01' |
| MG496810 | *Georychus capensis* | *Visser, Bennett & Jansen van Vuuren, 2018* | *Georychus capensis* | *Georychus capensis* | Citrusdal, South Africa | S 32°36'; E 19°01' |
| MG496758 | *Georychus capensis* | *Visser, Bennett & Jansen van Vuuren, 2018* | *Georychus capensis* | *Georychus capensis* | Moorreesburg, South Africa | S 33°17'; E 18°34' |
| MG496890 | *Georychus capensis* | *Visser, Bennett & Jansen van Vuuren, 2018* | *Georychus capensis* | *Georychus capensis* | Moorreesburg, South Africa | S 33°17'; E 18°34' |
| MG496715 | *Georychus capensis* | *Visser, Bennett & Jansen van Vuuren, 2018* | *Georychus capensis* | *Georychus capensis* | Moorreesburg, South Africa | S 33°17'; E 18°34' |
| MG496889 | *Georychus capensis* | *Visser, Bennett & Jansen van Vuuren, 2018* | *Georychus capensis* | *Georychus capensis* | Darling, South Africa | S 33°24'; E 18°24' |
| MG496749 | *Georychus capensis* | *Visser, Bennett & Jansen van Vuuren, 2018* | *Georychus capensis* | *Georychus capensis* | Darling, South Africa | S 33°24'; E 18°24' |
| MG496793 | *Georychus capensis* | *Visser, Bennett & Jansen van Vuuren, 2018* | *Georychus capensis* | *Georychus capensis* | Darling, South Africa | S 33°24'; E 18°24' |
| MG496752 | *Georychus capensis* | *Visser, Bennett & Jansen van Vuuren, 2018* | *Georychus capensis* | *Georychus capensis* | Darling, South Africa | S 33°24'; E 18°24' |
| MG496755 | *Georychus capensis* | *Visser, Bennett & Jansen van Vuuren, 2018* | *Georychus capensis* | *Georychus capensis* | Darling, South Africa | S 33°24'; E 18°24' |
| MG496756 | *Georychus capensis* | *Visser, Bennett & Jansen van Vuuren, 2018* | *Georychus capensis* | *Georychus capensis* | Darling, South Africa | S 33°24'; E 18°24' |
| MG496757 | *Georychus capensis* | *Visser, Bennett & Jansen van Vuuren, 2018* | *Georychus capensis* | *Georychus capensis* | Darling, South Africa | S 33°24'; E 18°24' |
| MG496717 | *Georychus capensis* | *Visser, Bennett & Jansen van Vuuren, 2018* | *Georychus capensis* | *Georychus capensis* | Cape Town, South Africa | S 34°00'; E 18°31' |
| MG496777 | *Georychus capensis* | *Visser, Bennett & Jansen van Vuuren, 2018* | *Georychus capensis* | *Georychus capensis* | Cape Town, South Africa | S 34°00'; E 18°31' |
| MG496705 | *Georychus capensis* | *Visser, Bennett & Jansen van Vuuren, 2018* | *Georychus capensis* | *Georychus capensis* | Cape Town, South Africa | S 34°00'; E 18°31' |
| MG496781 | *Georychus capensis* | *Visser, Bennett & Jansen van Vuuren, 2018* | *Georychus capensis* | *Georychus capensis* | Paarl, South Africa | S 33°44'; E 18°58' |
| MG496729 | *Georychus capensis* | *Visser, Bennett & Jansen van Vuuren, 2018* | *Georychus capensis* | *Georychus capensis* | Paarl, South Africa | S 33°44'; E 18°58' |
| MG496850 | *Georychus capensis* | *Visser, Bennett & Jansen van Vuuren, 2018* | *Georychus capensis* | *Georychus capensis* | Ceres, South Africa | S 33°12'; E 19°14' |
| MG496842 | *Georychus capensis* | *Visser, Bennett & Jansen van Vuuren, 2018* | *Georychus capensis* | *Georychus capensis* | Ceres, South Africa | S 33°12'; E 19°14' |
| MG496896 | *Georychus capensis* | *Visser, Bennett & Jansen van Vuuren, 2018* | *Georychus capensis* | *Georychus capensis* | Ceres, South Africa | S 33°12'; E 19°14' |
| MG496861 | *Georychus capensis* | *Visser, Bennett & Jansen van Vuuren, 2018* | *Georychus capensis* | *Georychus capensis* | Wolseley, South Africa | S 33°24'; E 19°12' |
| MG496841 | *Georychus capensis* | *Visser, Bennett & Jansen van Vuuren, 2018* | *Georychus capensis* | *Georychus capensis* | Wolseley, South Africa | S 33°24'; E 19°12' |
| MG496828 | *Georychus capensis* | *Visser, Bennett & Jansen van Vuuren, 2018* | *Georychus capensis* | *Georychus capensis* | Wolseley, South Africa | S 33°24'; E 19°12' |
| MG496831 | *Georychus capensis* | *Visser, Bennett & Jansen van Vuuren, 2018* | *Georychus capensis* | *Georychus capensis* | Wolseley, South Africa | S 33°24'; E 19°12' |
| MG496846 | *Georychus capensis* | *Visser, Bennett & Jansen van Vuuren, 2018* | *Georychus capensis* | *Georychus capensis* | Wolseley, South Africa | S 33°24'; E 19°12' |
| MG496893 | *Georychus capensis* | *Visser, Bennett & Jansen van Vuuren, 2018* | *Georychus capensis* | *Georychus capensis* | Wolseley, South Africa | S 33°24'; E 19°12' |
| MG496902 | *Georychus capensis* | *Visser, Bennett & Jansen van Vuuren, 2018* | *Georychus capensis* | *Georychus capensis* | Wolseley, South Africa | S 33°24'; E 19°12' |
| MG496864 | *Georychus capensis* | *Visser, Bennett & Jansen van Vuuren, 2018* | *Georychus capensis* | *Georychus capensis* | Wolseley, South Africa | S 33°24'; E 19°12' |
| MG496735 | *Georychus capensis* | *Visser, Bennett & Jansen van Vuuren, 2018* | *Georychus capensis* | *Georychus capensis* | Worcester, South Africa | S 33°40'; E 19°31' |
| MG496865 | *Georychus capensis* | *Visser, Bennett & Jansen van Vuuren, 2018* | *Georychus capensis* | *Georychus capensis* | Swellendam, South Africa | S 34°03'; E 20°25' |
| ***Bathyergus*** | | | | | | |
| **Accession number** | **Species designation: Public database** | **Original study** | **Species designation: Original study** | **Species designation: This study** | **Geographic information** | **Coordinates** |
| KJ866638 | *Bathyergus suillus* | *Visser, Bennett & Jansen van Vuuren, 2014* | *Bathyergus suillus* | *Bathyergus sp.1* | Struisbaai, South Africa | S 34°43'; E 20°00' |
| KJ866640 | *Bathyergus suillus* | *Visser, Bennett & Jansen van Vuuren, 2014* | *Bathyergus suillus* | *Bathyergus sp.1* | Struisbaai, South Africa | S 34°43'; E 20°00' |
| KJ866645 | *Bathyergus suillus* | *Visser, Bennett & Jansen van Vuuren, 2014* | *Bathyergus suillus* | *Bathyergus sp.1* | Struisbaai, South Africa | S 34°43'; E 20°00' |
| KJ866651 | *Bathyergus suillus* | *Visser, Bennett & Jansen van Vuuren, 2014* | *Bathyergus suillus* | *Bathyergus sp.1* | Struisbaai, South Africa | S 34°43'; E 20°00' |
| KJ866654 | *Bathyergus suillus* | *Visser, Bennett & Jansen van Vuuren, 2014* | *Bathyergus suillus* | *Bathyergus sp.1* | Struisbaai, South Africa | S 34°43'; E 20°00' |
| KJ866681 | *Bathyergus suillus* | *Visser, Bennett & Jansen van Vuuren, 2014* | *Bathyergus suillus* | *Bathyergus sp.2* | Sedgefield, South Africa | S 34°01'; E 22°48' |
| KJ866658 | *Bathyergus suillus* | *Visser, Bennett & Jansen van Vuuren, 2014* | *Bathyergus suillus* | *Bathyergus sp.2* | Riversdale, South Africa | S 34°10'; E 21°24' |
| KJ866659 | *Bathyergus suillus* | *Visser, Bennett & Jansen van Vuuren, 2014* | *Bathyergus suillus* | *Bathyergus sp.2* | Riversdale, South Africa | S 34°10'; E 21°24' |
| KJ866663 | *Bathyergus suillus* | *Visser, Bennett & Jansen van Vuuren, 2014* | *Bathyergus suillus* | *Bathyergus sp.2* | Riversdale, South Africa | S 34°10'; E 21°24' |
| KJ866664 | *Bathyergus suillus* | *Visser, Bennett & Jansen van Vuuren, 2014* | *Bathyergus suillus* | *Bathyergus sp.2* | Riversdale, South Africa | S 34°10'; E 21°24' |
| KJ866677 | *Bathyergus suillus* | *Visser, Bennett & Jansen van Vuuren, 2014* | *Bathyergus suillus* | *Bathyergus sp.2* | Riversdale, South Africa | S 34°10'; E 21°24' |
| MH186532 | *Bathyergus janetta* | *Visser, Bennett & Jansen van Vuuren, 2014* | *Bathyergus janetta* | *Bathyergus janetta* | Kamieskroon, South Africa | S 30°15'; E 17°53' |
| MH186533 | *Bathyergus janetta* | *Visser, Bennett & Jansen van Vuuren, 2014* | *Bathyergus janetta* | *Bathyergus janetta* | Kamieskroon, South Africa | S 30°15'; E 17°53' |
| KJ866626 | *Bathyergus suillus* | *Visser, Bennett & Jansen van Vuuren, 2014* | *Bathyergus suillus* | *Bathyergus suillus* | Stanford, South Africa | S 34°30'; E 19°27' |
| KJ866627 | *Bathyergus suillus* | *Visser, Bennett & Jansen van Vuuren, 2014* | *Bathyergus suillus* | *Bathyergus suillus* | Stanford, South Africa | S 34°30'; E 19°27' |
| KJ866630 | *Bathyergus suillus* | *Visser, Bennett & Jansen van Vuuren, 2014* | *Bathyergus suillus* | *Bathyergus suillus* | Stanford, South Africa | S 34°30'; E 19°27' |
| KJ866632 | *Bathyergus suillus* | *Visser, Bennett & Jansen van Vuuren, 2014* | *Bathyergus suillus* | *Bathyergus suillus* | Stanford, South Africa | S 34°30'; E 19°27' |
| KJ866608 | *Bathyergus suillus* | *Visser, Bennett & Jansen van Vuuren, 2014* | *Bathyergus suillus* | *Bathyergus suillus* | Cape Town, South Africa | S 33°55'; E 18°36' |
| KJ866609 | *Bathyergus suillus* | *Visser, Bennett & Jansen van Vuuren, 2014* | *Bathyergus suillus* | *Bathyergus suillus* | Cape Town, South Africa | S 33°55'; E 18°36' |
| KJ866610 | *Bathyergus suillus* | *Visser, Bennett & Jansen van Vuuren, 2014* | *Bathyergus suillus* | *Bathyergus suillus* | Cape Town, South Africa | S 33°55'; E 18°36' |
| KJ866611 | *Bathyergus suillus* | *Visser, Bennett & Jansen van Vuuren, 2014* | *Bathyergus suillus* | *Bathyergus suillus* | Cape Town, South Africa | S 33°55'; E 18°36' |
| KJ866612 | *Bathyergus suillus* | *Visser, Bennett & Jansen van Vuuren, 2014* | *Bathyergus suillus* | *Bathyergus suillus* | Cape Town, South Africa | S 33°55'; E 18°36' |
| KJ866614 | *Bathyergus suillus* | *Visser, Bennett & Jansen van Vuuren, 2014* | *Bathyergus suillus* | *Bathyergus suillus* | Cape Town, South Africa | S 33°55'; E 18°36' |
| KJ866617 | *Bathyergus suillus* | *Visser, Bennett & Jansen van Vuuren, 2014* | *Bathyergus suillus* | *Bathyergus suillus* | Cape Town, South Africa | S 33°55'; E 18°36' |
| KJ866618 | *Bathyergus suillus* | *Visser, Bennett & Jansen van Vuuren, 2014* | *Bathyergus suillus* | *Bathyergus suillus* | Cape Town, South Africa | S 33°55'; E 18°36' |
| KJ866619 | *Bathyergus suillus* | *Visser, Bennett & Jansen van Vuuren, 2014* | *Bathyergus suillus* | *Bathyergus suillus* | Cape Town, South Africa | S 33°55'; E 18°36' |
| KJ866622 | *Bathyergus suillus* | *Visser, Bennett & Jansen van Vuuren, 2014* | *Bathyergus suillus* | *Bathyergus suillus* | Cape Town, South Africa | S 33°55'; E 18°36' |
| KJ866550 | *Bathyergus suillus* | *Visser, Bennett & Jansen van Vuuren, 2014* | *Bathyergus suillus* | *Bathyergus suillus* | Redelinghuys, South Africa | S 32°25'; E 18°35' |
| KJ866552 | *Bathyergus suillus* | *Visser, Bennett & Jansen van Vuuren, 2014* | *Bathyergus suillus* | *Bathyergus suillus* | Redelinghuys, South Africa | S 32°25'; E 18°35' |
| KJ866558 | *Bathyergus suillus* | *Visser, Bennett & Jansen van Vuuren, 2014* | *Bathyergus suillus* | *Bathyergus suillus* | Redelinghuys, South Africa | S 32°25'; E 18°35' |
| KJ866561 | *Bathyergus suillus* | *Visser, Bennett & Jansen van Vuuren, 2014* | *Bathyergus suillus* | *Bathyergus suillus* | Redelinghuys, South Africa | S 32°25'; E 18°35' |
| KJ866562 | *Bathyergus suillus* | *Visser, Bennett & Jansen van Vuuren, 2014* | *Bathyergus suillus* | *Bathyergus suillus* | Redelinghuys, South Africa | S 32°25'; E 18°35' |
| KJ866563 | *Bathyergus suillus* | *Visser, Bennett & Jansen van Vuuren, 2014* | *Bathyergus suillus* | *Bathyergus suillus* | Redelinghuys, South Africa | S 32°25'; E 18°35' |
| KJ866564 | *Bathyergus suillus* | *Visser, Bennett & Jansen van Vuuren, 2014* | *Bathyergus suillus* | *Bathyergus suillus* | Redelinghuys, South Africa | S 32°25'; E 18°35' |
| KJ866569 | *Bathyergus suillus* | *Visser, Bennett & Jansen van Vuuren, 2014* | *Bathyergus suillus* | *Bathyergus suillus* | Dwarskersbos, South Africa | S 32°42'; E 18°20' |
| KJ866570 | *Bathyergus suillus* | *Visser, Bennett & Jansen van Vuuren, 2014* | *Bathyergus suillus* | *Bathyergus suillus* | Dwarskersbos, South Africa | S 32°42'; E 18°20' |
| KJ866571 | *Bathyergus suillus* | *Visser, Bennett & Jansen van Vuuren, 2014* | *Bathyergus suillus* | *Bathyergus suillus* | Dwarskersbos, South Africa | S 32°42'; E 18°20' |
| KJ866572 | *Bathyergus suillus* | *Visser, Bennett & Jansen van Vuuren, 2014* | *Bathyergus suillus* | *Bathyergus suillus* | Dwarskersbos, South Africa | S 32°42'; E 18°20' |
| KJ866575 | *Bathyergus suillus* | *Visser, Bennett & Jansen van Vuuren, 2014* | *Bathyergus suillus* | *Bathyergus suillus* | Dwarskersbos, South Africa | S 32°42'; E 18°20' |
| KJ866577 | *Bathyergus suillus* | *Visser, Bennett & Jansen van Vuuren, 2014* | *Bathyergus suillus* | *Bathyergus suillus* | Dwarskersbos, South Africa | S 32°42'; E 18°20' |
| KJ866582 | *Bathyergus suillus* | *Visser, Bennett & Jansen van Vuuren, 2014* | *Bathyergus suillus* | *Bathyergus suillus* | Dwarskersbos, South Africa | S 32°42'; E 18°20' |
| KJ866583 | *Bathyergus suillus* | *Visser, Bennett & Jansen van Vuuren, 2014* | *Bathyergus suillus* | *Bathyergus suillus* | Dwarskersbos, South Africa | S 32°42'; E 18°20' |
| KJ866510 | *Bathyergus suillus* | *Visser, Bennett & Jansen van Vuuren, 2014* | *Bathyergus suillus* | *Bathyergus suillus* | Sterkfontein, South Africa | S 32°47'; E 18°34' |
| KJ866511 | *Bathyergus suillus* | *Visser, Bennett & Jansen van Vuuren, 2014* | *Bathyergus suillus* | *Bathyergus suillus* | Sterkfontein, South Africa | S 32°47'; E 18°34' |
| KJ866512 | *Bathyergus suillus* | *Visser, Bennett & Jansen van Vuuren, 2014* | *Bathyergus suillus* | *Bathyergus suillus* | Sterkfontein, South Africa | S 32°47'; E 18°34' |
| KJ866513 | *Bathyergus suillus* | *Visser, Bennett & Jansen van Vuuren, 2014* | *Bathyergus suillus* | *Bathyergus suillus* | Sterkfontein, South Africa | S 32°47'; E 18°34' |
| KJ866522 | *Bathyergus suillus* | *Visser, Bennett & Jansen van Vuuren, 2014* | *Bathyergus suillus* | *Bathyergus suillus* | Sterkfontein, South Africa | S 32°47'; E 18°34' |
| KJ866525 | *Bathyergus suillus* | *Visser, Bennett & Jansen van Vuuren, 2014* | *Bathyergus suillus* | *Bathyergus suillus* | Sterkfontein, South Africa | S 32°47'; E 18°34' |
| KJ866526 | *Bathyergus suillus* | *Visser, Bennett & Jansen van Vuuren, 2014* | *Bathyergus suillus* | *Bathyergus suillus* | Sterkfontein, South Africa | S 32°47'; E 18°34' |
| KJ866589 | *Bathyergus suillus* | *Visser, Bennett & Jansen van Vuuren, 2014* | *Bathyergus suillus* | *Bathyergus suillus* | Piketberg, South Africa | S 32°54'; E 18°45' |
| KJ866590 | *Bathyergus suillus* | *Visser, Bennett & Jansen van Vuuren, 2014* | *Bathyergus suillus* | *Bathyergus suillus* | Piketberg, South Africa | S 32°54'; E 18°45' |
| KJ866530 | *Bathyergus suillus* | *Visser, Bennett & Jansen van Vuuren, 2014* | *Bathyergus suillus* | *Bathyergus suillus* | Vredenburg, South Africa | S 32°54'; E 18°01' |
| KJ866531 | *Bathyergus suillus* | *Visser, Bennett & Jansen van Vuuren, 2014* | *Bathyergus suillus* | *Bathyergus suillus* | Vredenburg, South Africa | S 32°54'; E 18°01' |
| KJ866534 | *Bathyergus suillus* | *Visser, Bennett & Jansen van Vuuren, 2014* | *Bathyergus suillus* | *Bathyergus suillus* | Vredenburg, South Africa | S 32°54'; E 18°01' |
| KJ866539 | *Bathyergus suillus* | *Visser, Bennett & Jansen van Vuuren, 2014* | *Bathyergus suillus* | *Bathyergus suillus* | Vredenburg, South Africa | S 32°54'; E 18°01' |
| KJ866541 | *Bathyergus suillus* | *Visser, Bennett & Jansen van Vuuren, 2014* | *Bathyergus suillus* | *Bathyergus suillus* | Vredenburg, South Africa | S 32°54'; E 18°01' |
| KJ866542 | *Bathyergus suillus* | *Visser, Bennett & Jansen van Vuuren, 2014* | *Bathyergus suillus* | *Bathyergus suillus* | Vredenburg, South Africa | S 32°54'; E 18°01' |
| KJ866543 | *Bathyergus suillus* | *Visser, Bennett & Jansen van Vuuren, 2014* | *Bathyergus suillus* | *Bathyergus suillus* | Vredenburg, South Africa | S 32°54'; E 18°01' |
| KJ866545 | *Bathyergus suillus* | *Visser, Bennett & Jansen van Vuuren, 2014* | *Bathyergus suillus* | *Bathyergus suillus* | Vredenburg, South Africa | S 32°54'; E 18°01' |
| KJ866548 | *Bathyergus suillus* | *Visser, Bennett & Jansen van Vuuren, 2014* | *Bathyergus suillus* | *Bathyergus suillus* | Vredenburg, South Africa | S 32°54'; E 18°01' |
| MH186540 | *Bathyergus suillus* | *Visser, Bennett & Jansen van Vuuren, 2019* | *Bathyergus suillus* | *Bathyergus suillus* | Moorreesburg, South Africa | S 33°17'; E 18°34' |
| MH186541 | *Bathyergus suillus* | *Visser, Bennett & Jansen van Vuuren, 2019* | *Bathyergus suillus* | *Bathyergus suillus* | Moorreesburg, South Africa | S 33°17'; E 18°34' |
| MH186536 | *Bathyergus suillus* | *Visser, Bennett & Jansen van Vuuren, 2019* | *Bathyergus suillus* | *Bathyergus suillus* | Klawer, South Africa | S 31°44'; E 16°36' |
| MH186537 | *Bathyergus suillus* | *Visser, Bennett & Jansen van Vuuren, 2019* | *Bathyergus suillus* | *Bathyergus suillus* | Klawer, South Africa | S 31°44'; E 16°36' |
| MH186538 | *Bathyergus suillus* | *Visser, Bennett & Jansen van Vuuren, 2019* | *Bathyergus suillus* | *Bathyergus suillus* | Klawer, South Africa | S 31°44'; E 16°36' |
| MH186534 | *Bathyergus suillus* | *Visser, Bennett & Jansen van Vuuren, 2019* | *Bathyergus suillus* | *Bathyergus suillus* | Lamberts Bay, South Africa | S 32°05'; E 18°22' |
| MH186535 | *Bathyergus suillus* | *Visser, Bennett & Jansen van Vuuren, 2019* | *Bathyergus suillus* | *Bathyergus suillus* | Lamberts Bay, South Africa | S 32°05'; E 18°22' |
| MH186539 | *Bathyergus suillus* | *Visser, Bennett & Jansen van Vuuren, 2019* | *Bathyergus suillus* | *Bathyergus suillus* | Gifberg, South Africa | S 31°49'; E 18°49' |
| ***Cryptomys*** | | | | | | |
| **Accession number** | **Species designation: Public database** | **Original study** | **Species designation: Original study** | **Species designation: This study** | **Geographic information** | **Coordinates** |
| AY425873 | *Cryptomys hottentotus mahali* | *Faulkes et al., 2004* | *Cryptomys hottentotus mahali* | *Cryptomys hottentotus mahali* | Patryshoek, Pretoria, South Africa | S 25°40'; E 28°02' |
| MH186551 | *Cryptomys hottentotus* | *Visser, Bennett & Jansen van Vuuren, 2019* | *Cryptomys hottentotus mahali* | *Cryptomys hottentotus mahali* | Swellendam, South Africa | S 34°03'; E 20°25' |
| MH186552 | *Cryptomys hottentotus* | *Visser, Bennett & Jansen van Vuuren, 2019* | *Cryptomys hottentotus mahali* | *Cryptomys hottentotus mahali* | Swellendam, South Africa | S 34°03'; E 20°25' |
| MH186553 | *Cryptomys hottentotus* | *Visser, Bennett & Jansen van Vuuren, 2019* | *Cryptomys hottentotus mahali* | *Cryptomys hottentotus mahali* | Heidelberg, South Africa | S 34°05'; E 20°57' |
| MH186554 | *Cryptomys hottentotus* | *Visser, Bennett & Jansen van Vuuren, 2019* | *Cryptomys hottentotus mahali* | *Cryptomys hottentotus mahali* | Heidelberg, South Africa | S 34°05'; E 20°57' |
| MH186555 | *Cryptomys hottentotus* | *Visser, Bennett & Jansen van Vuuren, 2019* | *Cryptomys hottentotus mahali* | *Cryptomys hottentotus mahali* | Struisbaai, South Africa | S 34°41'; E 20°00' |
| AF012237 | *Cryptomys hottentotus nimrodi* | *Faulkes et al., 2004* | *Cryptomys hottentotus nimrodi* | *Cryptomys hottentotus nimrodi* | Hillside, Zimbabwe | S 20°55'; E 28°38' |
| AY425885 | *Cryptomys hottentotus nimrodi* | *Faulkes et al., 2004* | *Cryptomys hottentotus nimrodi* | *Cryptomys hottentotus nimrodi* | Limpopo Valley, Zimbabwe | S 22°30'; E 28°40' |
| AY425886 | *Cryptomys hottentotus nimrodi* | *Faulkes et al., 2004* | *Cryptomys hottentotus nimrodi* | *Cryptomys hottentotus nimrodi* | Bulawayo, Zimbabwe | S 20°09'; E 28°38' |
| AY425888 | *Cryptomys hottentotus nimrodi* | *Faulkes et al., 2004* | *Cryptomys hottentotus nimrodi* | *Cryptomys hottentotus nimrodi* | Bulawayo, Zimbabwe | S 20°09'; E 28°38' |
| AF012238 | *Cryptomys hottentotus hottentotus* | *Faulkes et al., 2004* | *Cryptomys hottentotus hottentotus* | *Cryptomys hottentotus hottentotus* | Klawer, South Africa | S 31°48'; E 18°38' |
| AF012239 | *Cryptomys hottentotus hottentotus* | *Faulkes et al., 2004* | *Cryptomys hottentotus hottentotus* | *Cryptomys hottentotus hottentotus* | Somerset West, South Africa | S 34°04'; E 18°50' |
| AF012240 | *Cryptomys hottentotus hottentotus* | *Faulkes et al., 2004* | *Cryptomys hottentotus hottentotus* | *Cryptomys hottentotus hottentotus* | Steinkopf, South Africa | S 29°17'; E 17°45' |
| MH186560 | *Cryptomys hottentotus* | *Visser, Bennett & Jansen van Vuuren, 2019* | *Cryptomys hottentotus hottentotus* | *Cryptomys hottentotus hottentotus* | Darling, South Africa | S 33°24'; E 18°24' |
| MH186557 | *Cryptomys hottentotus* | *Visser, Bennett & Jansen van Vuuren, 2019* | *Cryptomys hottentotus hottentotus* | *Cryptomys hottentotus hottentotus* | Moorreesburg, South Africa | S 33°17'; E 18°34' |
| MH186558 | *Cryptomys hottentotus* | *Visser, Bennett & Jansen van Vuuren, 2019* | *Cryptomys hottentotus hottentotus* | *Cryptomys hottentotus hottentotus* | Moorreesburg, South Africa | S 33°17'; E 18°34' |
| MH186559 | *Cryptomys hottentotus* | *Visser, Bennett & Jansen van Vuuren, 2019* | *Cryptomys hottentotus hottentotus* | *Cryptomys hottentotus hottentotus* | Moorreesburg, South Africa | S 33°17'; E 18°34' |
| MH186556 | *Cryptomys hottentotus* | *Visser, Bennett & Jansen van Vuuren, 2019* | *Cryptomys hottentotus hottentotus* | *Cryptomys hottentotus hottentotus* | Paarl, South Africa | S 33°44'; E 18°58' |
| MH186548 | *Cryptomys hottentotus* | *Visser, Bennett & Jansen van Vuuren, 2019* | *Cryptomys hottentotus hottentotus* | *Cryptomys hottentotus hottentotus* | Oudshoorn, South Africa | S 33°51'; E 22°02' |
| MH186549 | *Cryptomys hottentotus* | *Visser, Bennett & Jansen van Vuuren, 2019* | *Cryptomys hottentotus hottentotus* | *Cryptomys hottentotus hottentotus* | Oudshoorn, South Africa | S 33°51'; E 22°02' |
| MH186550 | *Cryptomys hottentotus* | *Visser, Bennett & Jansen van Vuuren, 2019* | *Cryptomys hottentotus hottentotus* | *Cryptomys hottentotus hottentotus* | Oudshoorn, South Africa | S 33°51'; E 22°02' |
| AF012235 | *Cryptomys hottentotus natalensis* | *Faulkes et al., 2004* | *Cryptomys hottentotus natalensis* | *Cryptomys hottentotus natalensis* | Kokstad, South Africa | S 31°32'; E 29°38' |
| AY425869 | *Cryptomys hottentotus natalensis* | *Faulkes et al., 2004* | *Cryptomys hottentotus natalensis* | *Cryptomys hottentotus natalensis* | Komatiepoort, South Africa | S 25°25'; E 31°57' |
| MH186542 | *Cryptomys hottentotus* | *Visser, Bennett & Jansen van Vuuren, 2019* | *Cryptomys hottentotus natalensis* | *Cryptomys hottentotus natalensis* | Wakkerstroom, South Africa | S 27°18'; E 30°16' |
| MH186543 | *Cryptomys hottentotus* | *Visser, Bennett & Jansen van Vuuren, 2019* | *Cryptomys hottentotus natalensis* | *Cryptomys hottentotus natalensis* | Nottingham Road, South Africa | S 29°29'; E 29°52' |
| MH186544 | *Cryptomys hottentotus* | *Visser, Bennett & Jansen van Vuuren, 2019* | *Cryptomys hottentotus natalensis* | *Cryptomys hottentotus natalensis* | Nottingham Road, South Africa | S 29°29'; E 29°52' |
| MH186545 | *Cryptomys hottentotus* | *Visser, Bennett & Jansen van Vuuren, 2019* | *Cryptomys hottentotus natalensis* | *Cryptomys hottentotus natalensis* | Nottingham Road, South Africa | S 29°29'; E 29°52' |
| MH186546 | *Cryptomys hottentotus* | *Visser, Bennett & Jansen van Vuuren, 2019* | *Cryptomys hottentotus natalensis* | *Cryptomys hottentotus natalensis* | Nottingham Road, South Africa | S 29°29'; E 29°52' |
| MH186547 | *Cryptomys hottentotus* | *Visser, Bennett & Jansen van Vuuren, 2019* | *Cryptomys hottentotus natalensis* | *Cryptomys hottentotus natalensis* | Nottingham Road, South Africa | S 29°29'; E 29°52' |
| AF012236 | *Cryptomys hottentotus pretoriae* | *Faulkes et al., 2004* | *Cryptomys hottentotus pretoriae* | *Cryptomys hottentotus pretoriae* | Hekpoort, South Africa | S 25°52'; E 27°37' |
| AY425874 | *Cryptomys hottentotus pretoriae* | *Faulkes et al., 2004* | *Cryptomys hottentotus pretoriae* | *Cryptomys hottentotus pretoriae* | Hekpoort, South Africa | S 25°52'; E 27°37' |
| AY425882 | *Cryptomys hottentotus pretoriae* | *Faulkes et al., 2004* | *Cryptomys hottentotus pretoriae* | *Cryptomys hottentotus pretoriae* | Pretoria, South Africa | S 25°47'; E 28°13' |
| AY425883 | *Cryptomys hottentotus pretoriae* | *Faulkes et al., 2004* | *Cryptomys hottentotus pretoriae* | *Cryptomys hottentotus pretoriae* | Krugersdorp, South Africa | S 26°06'; E 27°43' |
| AY425884 | *Cryptomys hottentotus pretoriae* | *Faulkes et al., 2004* | *Cryptomys hottentotus pretoriae* | *Cryptomys hottentotus pretoriae* | Krugersdorp, South Africa | S 26°06'; E 27°43' |
| AY425875 | *Cryptomys hottentotus pretoriae* | *Faulkes et al., 2004* | *Cryptomys hottentotus pretoriae* | *Cryptomys hottentotus pretoriae* | Johannesburg, South Africa | S 26°11'; E 28°04' |
| AY425876 | *Cryptomys hottentotus pretoriae* | *Faulkes et al., 2004* | *Cryptomys hottentotus pretoriae* | *Cryptomys hottentotus pretoriae* | Johannesburg, South Africa | S 26°11'; E 28°04' |
| AY425877 | *Cryptomys hottentotus pretoriae* | *Faulkes et al., 2004* | *Cryptomys hottentotus pretoriae* | *Cryptomys hottentotus pretoriae* | Johannesburg, South Africa | S 26°11'; E 28°04' |
| AY425878 | *Cryptomys hottentotus pretoriae* | *Faulkes et al., 2004* | *Cryptomys hottentotus pretoriae* | *Cryptomys hottentotus pretoriae* | Johannesburg, South Africa | S 26°11'; E 28°04' |
| AY425879 | *Cryptomys hottentotus pretoriae* | *Faulkes et al., 2004* | *Cryptomys hottentotus pretoriae* | *Cryptomys hottentotus pretoriae* | Pretoria, South Africa | S 25°47'; E 28°13' |
| AY425880 | *Cryptomys hottentotus pretoriae* | *Faulkes et al., 2004* | *Cryptomys hottentotus pretoriae* | *Cryptomys hottentotus pretoriae* | Pretoria, South Africa | S 25°47'; E 28°13' |
| AY425881 | *Cryptomys hottentotus pretoriae* | *Faulkes et al., 2004* | *Cryptomys hottentotus pretoriae* | *Cryptomys hottentotus pretoriae* | Pretoria, South Africa | S 25°47'; E 28°13' |
| ***Fukomys*** | | | | | | |
| **Accession number** | **Species designation: Public database** | **Original study** | **Species designation: Original study** | **Species designation: This study** | **Geographic information** | **Coordinates** |
| KX905198 | *Fukomys zechi* | - | - | *Fukomys zechi* | *-* | - |
| AF012229 | *Cryptomys bocagei* | *Faulkes et al., 2004* | *Cryptomys bocagei* | *Fukomys bocagei* | Lubango, Angola | S 14°56'; E 13°27' |
| AY425864 | *Cryptomys mechowi* | *Faulkes et al., 2004* | *Cryptomys mechowii* | *Fukomys mechowii* | Kapiri Mposhi, Zambia | S 13°58'; E 28°40' |
| AY425865 | *Cryptomys mechowi* | *Faulkes et al., 2004* | *Cryptomys mechowii* | *Fukomys mechowii* | Kapiri Mposhi, Zambia | S 13°58'; E 28°40' |
| AY425867 | *Cryptomys mechowi* | *Faulkes et al., 2004* | *Cryptomys mechowii* | *Fukomys mechowii* | Chingola, Zambia | S 12°31'; E 27°51' |
| AY425868 | *Cryptomys mechowi* | *Faulkes et al., 2004* | *Cryptomys mechowii* | *Fukomys mechowii* | Chingola, Zambia | S 12°31'; E 27°51' |
| AF012230 | *Cryptomys mechowi* | *Faulkes et al., 2004* | *Cryptomys mechowii* | *Fukomys mechowii* | Chingola, Zambia | S 12°31'; E 27°51' |
| AF012231 | *Cryptomys mechowi* | *Faulkes et al., 2004* | *Cryptomys mechowii* | *Fukomys mechowii* | Kinshasa, DRC | S 04°22'; E 15°27' |
| EF043451 | *Fukomys mechowii* | *Van Daele et al., 2007b* | *Fukomys mechowii* | *Fukomys mechowii* | Chiundaponde, Zambia | S 12°14'; E 30°35' |
| EF043452 | *Fukomys mechowii* | *Van Daele et al., 2007b* | *Fukomys mechowii* | *Fukomys mechowii* | Chibale, Zambia | S 13°35'; E 30°05' |
| EF043455 | *Fukomys sp.* | *Van Daele et al., 2007b* | *Fukomys mechowii* | *Fukomys mechowii* | Salujinga, Zambia | S 10°58'; E 24°05' |
| KX905196 | *Fukomys sp.* | *Faulkes et al., 2017* | *Fukomys livingstoni* | *Fukomys livingstoni* | Ujiji, Tanzania | S 04°52'; E 28°42' |
| KX905197 | *Fukomys sp.* | *Faulkes et al., 2017* | *Fukomys livingstoni* | *Fukomys livingstoni* | Ujiji, Tanzania | S 04°52'; E 29°42' |
| AY425863 | *Cryptomys whytei* | *Faulkes et al., 2004* | *Cryptomys darlingi* | *Fukomys hanangensis* | Mzuzu, Malawi | S 11°27'; E 34°03' |
| GU197595 | *Fukomys sp.* | *Faulkes et al., 2017* | *Fukomys hanangensis* | *Fukomys hanangensis* | Hanang, Tanzania | S 04°24'; E 35°27' |
| GU197596 | *Fukomys sp.* | *Faulkes et al., 2017* | *Fukomys hanangensis* | *Fukomys hanangensis* | Hanang, Tanzania | S 04°24'; E 35°27' |
| KX905191 | *Fukomys sp.* | *Faulkes et al., 2017* | *Fukomys hanangensis* | *Fukomys hanangensis* | Mbulu, Tanzania | S 04°03'; E 35°26' |
| EF043465 | *Fukomys amatus* | *Van Daele et al., 2007b* | *Fukomys amatus* | *Fukomys amatus* | Kama, Zambia | S 12°24'; E 30°21' |
| EF043467 | *Fukomys amatus* | *Van Daele et al., 2007b* | *Fukomys amatus* | *Fukomys amatus* | Chinsobwe, Zambia | S 13°23'; E 30°21' |
| EF043468 | *Fukomys amatus* | *Van Daele et al., 2007b* | *Fukomys amatus* | *Fukomys amatus* | Chibale, Zambia | S 13°35'; E 30°05' |
| EF043458 | *Fukomys sp.* | *Van Daele et al., 2007b* | *Fukomys whytei* | *Fukomys Sp.1* | Kasanka, Zambia | S 12°08'; E 29°47' |
| EF043459 | *Fukomys sp.* | *Van Daele et al., 2007b* | *Fukomys whytei* | *Fukomys Sp.1* | Kasanka, Zambia | S 12°08'; E 29°47' |
| EF043460 | *Fukomys sp.* | *Van Daele et al., 2007b* | *Fukomys whytei* | *Fukomys Sp.1* | Kasanka, Zambia | S 12°08'; E 29°47' |
| EF043461 | *Fukomys sp.* | *Van Daele et al., 2007b* | *Fukomys whytei* | *Fukomys Sp.1* | Kambi, Zambia | S 11°30'; E 29°34' |
| EF043463 | *Fukomys sp.* | *Van Daele et al., 2007b* | *Fukomys whytei* | *Fukomys Sp.1* | Kakululu River, Zambia | S 10°38'; E 29°04' |
| EF043464 | *Fukomys sp.* | *Van Daele et al., 2007b* | *Fukomys whytei* | *Fukomys Sp.1* | Chief Tungati Local Forest, Zambia | S 10°27'; E 30°00' |
| AY425859 | *Cryptomys whytei* | *Faulkes et al., 2004* | *Cryptomys whytei* | *Fukomys whytei* | Suma, Tanzania | S 09°10'; E 33°40' |
| AY425860 | *Cryptomys whytei* | *Faulkes et al., 2004* | *Cryptomys whytei* | *Fukomys whytei* | Mbala, Zambia | S 09°50'; E 31°24' |
| AY425861 | *Cryptomys whytei* | *Faulkes et al., 2004* | *Cryptomys whytei* | *Fukomys whytei* | Mbala, Zambia | S 09°50'; E 31°24' |
| AY425862 | *Cryptomys whytei* | *Faulkes et al., 2004* | *Cryptomys whytei* | *Fukomys whytei* | Mbala, Zambia | S 09°50'; E 31°24' |
| EF043477 | *Fukomys whytei* | *Van Daele et al., 2007b* | *Fukomys whytei* | *Fukomys whytei* | Karonga, Malawi | S 09°56'; E 33°56' |
| GU197601 | *Fukomys whytei occlusus* | *Faulkes et al., 2010* | *Fukomys sp.* | *Fukomys w. occlusus* | Kigogo, Tanzania | S 08°38'; E 35°12' |
| EF043469 | *Fukomys sp.* | *Van Daele et al., 2007b* | *Fukomys whytei* | *Fukomys Sp.2* | Mushangashi, Zambia | S 12°28'; E 30°23' |
| EF043470 | *Fukomys sp.* | *Van Daele et al., 2007b* | *Fukomys whytei* | *Fukomys Sp.2* | Lake Chiwakawaka, Zambia | S 12°32'; E 30°37' |
| EF043471 | *Fukomys sp.* | *Van Daele et al., 2007b* | *Fukomys whytei* | *Fukomys Sp.2* | Lake Chiwakawaka, Zambia | S 12°32'; E 30°37' |
| EF043472 | *Fukomys sp.* | *Van Daele et al., 2007b* | *Fukomys whytei* | *Fukomys Sp.2* | Ndeba, Zambia | S 12°26'; E 30°39' |
| EF043473 | *Fukomys sp.* | *Van Daele et al., 2007b* | *Fukomys whytei* | *Fukomys Sp.2* | Ndeba, Zambia | S 12°28'; E 30°38' |
| EF043474 | *Fukomys sp.* | *Van Daele et al., 2007b* | *Fukomys whytei* | *Fukomys Sp.2* | Kasama, Zambia | S 10°16'; E 31°00' |
| EF043475 | *Fukomys sp.* | *Van Daele et al., 2007b* | *Fukomys whytei* | *Fukomys Sp.2* | Lufubu, Zambia | S 09°15'; E 30°53' |
| EF043476 | *Fukomys sp.* | *Van Daele et al., 2007b* | *Fukomys whytei* | *Fukomys Sp.2* | Lufubu, Zambia | S 09°15'; E 30°53' |
| AF012232 | *Cryptomys darlingi* | *Faulkes et al., 2004* | *Cryptomys darlingi* | *Fukomys darlingi* | Goromonzi, Zimbabwe | S 17°52'; E 31°30' |
| AF012220 | *Cryptomys damarensis* | *Faulkes et al., 2004* | *Fukomys damarensis* | *Fukomys damarensis* | Okavango Delta, Botswana | S 19°32'; E 23°11' |
| AF012221 | *Cryptomys damarensis* | *Faulkes et al., 2004* | *Cryptomys damarensis* | *Fukomys damarensis* | Maun, Botswana | S 19°59'; E 23°21' |
| AF012222 | *Cryptomys damarensis* | *Faulkes et al., 2004* | *Cryptomys damarensis* | *Fukomys damarensis* | Maun, Botswana | S 19°59'; E 23°21' |
| AF012223 | *Cryptomys damarensis* | *Faulkes et al., 2004* | *Cryptomys damarensis* | *Fukomys damarensis* | Okavango Delta, Botswana | S 19°32'; E 23°11' |
| AF012224 | *Cryptomys damarensis* | *Faulkes et al., 2004* | *Cryptomys damarensis* | *Fukomys damarensis* | Okavango Delta, Botswana | S 19°32'; E 23°11' |
| AF012225 | *Cryptomys damarensis* | *Faulkes et al., 2004* | *Cryptomys damarensis* | *Fukomys damarensis* | Dorbabis, Namibia | S 22°58'; E 17°41' |
| AF012226 | *Cryptomys damarensis* | *Faulkes et al., 2004* | *Cryptomys damarensis* | *Fukomys damarensis* | Dorbabis, Namibia | S 22°58'; E 17°41' |
| AF012227 | *Cryptomys damarensis* | *Faulkes et al., 2004* | *Cryptomys damarensis* | *Fukomys damarensis* | Dorbabis, Namibia | S 22°58'; E 17°41' |
| AF012228 | *Cryptomys damarensis* | *Faulkes et al., 2004* | *Cryptomys damarensis* | *Fukomys damarensis* | Dorbabis, Namibia | S 22°58'; E 17°41' |
| AY425848 | *Cryptomys damarensis* | *Faulkes et al., 2004* | *Cryptomys damarensis* | *Fukomys damarensis* | Hotazel, South Africa | S 27°17'; E 23°00' |
| AY425849 | *Cryptomys damarensis* | *Faulkes et al., 2004* | *Cryptomys damarensis* | *Fukomys damarensis* | Hotazel, South Africa | S 27°17'; E 23°00' |
| AY425850 | *Cryptomys damarensis* | *Faulkes et al., 2004* | *Cryptomys damarensis* | *Fukomys damarensis* | Hotazel, South Africa | S 27°17'; E 23°00' |
| AY425851 | *Cryptomys damarensis* | *Faulkes et al., 2004* | *Cryptomys damarensis* | *Fukomys damarensis* | Hotazel, South Africa | S 27°17'; E 23°00' |
| AY425852 | *Cryptomys damarensis* | *Faulkes et al., 2004* | *Cryptomys damarensis* | *Fukomys damarensis* | Hotazel, South Africa | S 27°17'; E 23°00' |
| AY425853 | *Cryptomys damarensis* | *Faulkes et al., 2004* | *Cryptomys damarensis* | *Fukomys damarensis* | Hotazel, South Africa | S 27°17'; E 23°00' |
| AY425854 | *Cryptomys damarensis* | *Faulkes et al., 2004* | *Cryptomys damarensis* | *Fukomys damarensis* | Hotazel, South Africa | S 27°17'; E 23°00' |
| AY425855 | *Cryptomys damarensis* | *Faulkes et al., 2004* | *Cryptomys damarensis* | *Fukomys damarensis* | Hotazel, South Africa | S 27°17'; E 23°00' |
| AY425856 | *Cryptomys damarensis* | *Faulkes et al., 2004* | *Cryptomys damarensis* | *Fukomys damarensis* | Hotazel, South Africa | S 27°17'; E 23°00' |
| AY425857 | *Cryptomys damarensis* | *Faulkes et al., 2004* | *Cryptomys damarensis* | *Fukomys damarensis* | Bulawayo, Zimbabwe | S 20°09'; E 28°38' |
| AY425858 | *Cryptomys damarensis* | *Faulkes et al., 2004* | *Cryptomys damarensis* | *Fukomys damarensis* | Rundu, Namibia | S 17°48'; E 19°32' |
| U87526 | *Cryptomys damarensis* | *Faulkes et al., 2004* | *Cryptomys damarensis* | *Fukomys damarensis* | Okavango Delta, Botswana | S 19°32'; E 23°11' |
| EF043480 | *Fukomys sp.* | *Van Daele et al., 2007b* | *Fukomys damarensis* | *Fukomys damarensis* | Sioma Ngwezi N.P., Zambia | S 17°15'; E 23°25' |
| EF043481 | *Fukomys sp.* | *Van Daele et al., 2007b* | *Fukomys damarensis* | *Fukomys damarensis* | Sioma Ngwezi N.P., Zambia | S 17°15'; E 23°25' |
| EF043479 | *Fukomys sp.* | *Van Daele et al., 2007b* | *Fukomys damarensis* | *Fukomys damarensis* | Simungoma, Zambia | S 17°25'; E 24°45' |
| EF043482 | *Fukomys sp.* | *Van Daele et al., 2007b* | *Fukomys micklemi* | *Fukomys Sp.3* | Chinyingi, Zambia | S 13°23'; E 23°00' |
| EF043483 | *Fukomys sp.* | *Van Daele et al., 2007b* | *Fukomys micklemi* | *Fukomys Sp.3* | Mayau, Zambia | S 12°44'; E 24°20' |
| EF043484 | *Fukomys sp.* | *Van Daele et al., 2007b* | *Fukomys micklemi* | *Fukomys Sp.3* | Mayau, Zambia | S 12°44'; E 24°20' |
| EF043485 | *Fukomys sp.* | *Van Daele et al., 2007b* | *Fukomys micklemi* | *Fukomys Sp.3* | Watopa, Zambia | S 14°00'; E 23°47' |
| EF043486 | *Fukomys sp.* | *Van Daele et al., 2007b* | *Fukomys micklemi* | *Fukomys Sp.3* | Watopa, Zambia | S 14°00'; E 23°47' |
| AF012233 | *Cryptomys amatus* | *Faulkes et al., 2004* | *Cryptomys anselli* | *Fukomys anselli* | Lusaka, Zambia | S 15°19'; E 28°27' |
| EF043490 | *Fukomys sp.* | *Van Daele et al., 2007b* | *Fukomys micklemi* | *Fukomys anselli* | Munali, Zambia | S 15°58'; E 28°08' |
| EF043491 | *Fukomys sp.* | *Van Daele et al., 2007b* | *Fukomys micklemi* | *Fukomys anselli* | Moono, Zambia | S 15°08'; E 26°57' |
| EF043492 | *Fukomys sp.* | *Van Daele et al., 2007b* | *Fukomys micklemi* | *Fukomys anselli* | Kaindu, Zambia | S 14°29'; E 26°54' |
| EF043493 | *Fukomys micklemi* | *Van Daele et al., 2007b* | *Fukomys micklemi* | *Fukomys micklemi* | Kataba, Zambia | S 15°23'; E 23°23' |
| EF043494 | *Fukomys micklemi* | *Van Daele et al., 2007b* | *Fukomys micklemi* | *Fukomys micklemi* | Kataba, Zambia | S 15°23'; E 23°23' |
| EF043495 | *Fukomys sp.* | *Van Daele et al., 2007b* | *Fukomys micklemi* | *Fukomys micklemi* | Senanga, Zambia | S 15°58'; E 23°20' |
| EF043496 | *Fukomys sp.* | *Van Daele et al., 2007b* | *Fukomys micklemi* | *Fukomys micklemi* | Luampa, Zambia | S 15°03'; E 24°24' |
| AF012234 | *Cryptomys choma* | *Faulkes et al., 2004* | *Cryptomys sp.* | *Fukomys choma* | Kalomo, Zambia | S 16°45'; E 27°00' |
| EF043487 | *Fukomys sp.* | *Van Daele et al., 2007b* | *Fukomys micklemi* | *Fukomys choma* | Namwala N, Zambia | S 15°40'; E 26°25' |
| EF043497 | *Fukomys sp.* | *Van Daele et al., 2007b* | *Fukomys micklemi* | *Fukomys choma* | Mazabuka, Zambia | S 16°12'; E 27°25' |
| EF043498 | *Fukomys sp.* | *Van Daele et al., 2007b* | *Fukomys micklemi* | *Fukomys choma* | Monze, Zambia | S 16°04'; E 27°32' |
| EF043499 | *Fukomys sp.* | *Van Daele et al., 2007b* | *Fukomys micklemi* | *Fukomys choma* | Lochinvar, Zambia | S 16°06'; E 27°18' |
| EF043500 | *Fukomys sp.* | *Van Daele et al., 2007b* | *Fukomys micklemi* | *Fukomys choma* | Dongo, Zambia | S 16°38'; E 26°27' |
| EF043501 | *Fukomys sp.* | *Van Daele et al., 2007b* | *Fukomys micklemi* | *Fukomys choma* | Dongo, Zambia | S 16°38'; E 26°27' |
| EF043503 | *Fukomys sp.* | *Van Daele et al., 2007b* | *Fukomys micklemi* | *Fukomys kafuensis* | Kavumba, Zambia | S 17°35'; E 25°21' |
| EF043504 | *Fukomys sp.* | *Van Daele et al., 2007b* | *Fukomys micklemi* | *Fukomys kafuensis* | Kalamba, Zambia | S 17°38'; E 25°41' |
| EF043509 | *Fukomys sp.* | *Van Daele et al., 2007b* | *Fukomys micklemi* | *Fukomys kafuensis* | Ndrevu, Zambia | S 17°38'; E 25°41' |
| EF043510 | *Fukomys sp.* | *Van Daele et al., 2007b* | *Fukomys micklemi* | *Fukomys kafuensis* | Livingstone, Zambia | S 17°54'; E 25°53' |
| EF043512 | *Fukomys sp.* | *Van Daele et al., 2007b* | *Fukomys micklemi* | *Fukomys kafuensis* | Mikata, Zambia | - |
| EF043513 | *Fukomys sp.* | *Van Daele et al., 2007b* | *Fukomys micklemi* | *Fukomys kafuensis* | Kajunika lila, Zambia | - |
| EF043514 | *Fukomys sp.* | *Van Daele et al., 2007b* | *Fukomys micklemi* | *Fukomys kafuensis* | Kajunika lila, Zambia | - |
| EF043515 | *Fukomys sp.* | *Van Daele et al., 2007b* | *Fukomys micklemi* | *Fukomys kafuensis* | Kajunika lila, Zambia | - |
| EF043516 | *Fukomys sp.* | *Van Daele et al., 2007b* | *Fukomys kafuensis* | *Fukomys kafuensis* | Itezhi-Itezhi, Zambia | S 15°51'; E 26°03' |
| **Outgroups** | | | | | | |
| **Accession number** | **Species designation: Public database** | **Original study** | **Species designation: Original study** | **Species designation: This study** | **Geographic information** | **Coordinates** |
| MH186592 | *Hystrix africaeaustralis* | *Visser, Bennett & Jansen van Vuuren, 2019* | *Hystrix africaeaustralis* | *Hystrix africaeaustralis* | Moorreesburg, South Africa | S 33°17'; E 18°34' |
| MH186593 | *Hystrix africaeaustralis* | *Visser, Bennett & Jansen van Vuuren, 2019* | *Hystrix africaeaustralis* | *Hystrix africaeaustralis* | Oudshoorn, South Africa | S 33°51'; E 22°02' |
| MH186591 | *Petromus typicus* | *Visser, Bennett & Jansen van Vuuren, 2019* | *Petromus typicus* | *Petromus typicus* | Nuwerus, South Africa | S 31°10'; E 18°23' |
| KJ742647 | *Thryonomys swinderianus* | *Upham & Patterson, 2015* | *Thryonomys swinderianus* | *Thryonomys swinderianus* | *Upham & Patterson, 2015* | - |
