# Supplementary material for: Phylogeny and biogeography of the African Bathyergidae: a review of patterns and processes
Source: PeerJ. 2019 Oct 15;7:e7730. doi: 10.7717/peerj.7730 (PMC6798870; doi:10.7717/peerj.7730)
Supplement: Supplemental Information 3 [file peerj-07-7730-s003.docx]

| ***Species*** | **Authority** |
| --- | --- |
| ***Heterocephalus*** | |
| *H. glaber* | Rüppell, 1842 |
| ***Heliophobius*** | |
| *H. argenteocinereus* | [Peters, 1846](https://en.wikipedia.org/wiki/Wilhelm_Peters) |
| *H. emini* | Noack, 1894 |
| ***Georychus*** | |
| *G. capensis* | [Illiger, 1811](https://en.wikipedia.org/wiki/Johann_Karl_Wilhelm_Illiger) |
| ***Bathyergus*** | |
| *B. janetta* | [Thomas & Schwann, 1904](https://en.wikipedia.org/wiki/Oldfield_Thomas) |
| *B. suillus* | [Schreber, 1782](https://en.wikipedia.org/wiki/Johann_Christian_Daniel_von_Schreber) |
| ***Cryptomys*** | |
| *C. h. hottentotus* | Lesson, 1826 |
| *C. h. mahali* | Roberts, 1913 |
| *C. h. natalensis* | Roberts, 1913 |
| *C. h. nimrodi* | de Winton, 1896 |
| *C. h. pretoriae* | Roberts, 1913 |
| ***Fukomys*** | |
| *F. amatus* | Wroughton, 1907; Macholan et al. 1998 |
| *F. anselli* | Filippucci et al. 1994; Burda et al. 1999; Chitaukali et al. 2001 |
| *F. bocagei* | De Winton, 1897; Honeycutt et al. 1991 |
| *F. choma* | Faulkes et al. 1997 |
| *F. damarensis* | Ogilby, 1838; Honeycutt et al. 1991 |
| *F. darlingi* | Thomas, 1895; Aguilar, 1993 |
| *F. hanangensis* | Faulkes et al. 2017 |
| *F. kafuensis* | Filippucci et al. 1994; Burda et al. 1999 |
| *F. livingstoni* | Faulkes et al. 2017 |
| *F. mechowii* | Peters, 1881; Honeycutt et al. 1991 |
| *F. micklemi* | Chubb, 1909 |
| *F. whytei* | Thomas, 1897; Chitaukali et al. 2001 |
| *F. zechi* | Matschie, 1900; Honeycutt et al. 1991 |
