# Supplementary material for: Phylogeny and biogeography of the African Bathyergidae: a review of patterns and processes
Source: PeerJ. 2019 Oct 15;7:e7730. doi: 10.7717/peerj.7730 (PMC6798870; doi:10.7717/peerj.7730)
Supplement: Supplemental Information 4 — Pairwise estimates of uncorrected sequence divergence among the six bathyergid genera. [file peerj-07-7730-s004.docx]

| **Genus** | *Heterocephalus* | *Heliophobius* | *Georychus* | *Bathyergus* | *Cryptomys* | *Fukomys* |
| --- | --- | --- | --- | --- | --- | --- |
| *Heterocephalus* | - |  |  |  |  |  |
| *Heliophobius* | 25.5 | - |  |  |  |  |
| *Georychus* | 24.2 | 22.8 | - |  |  |  |
| *Bathyergus* | 21.6 | 21.7 | 16.5 | - |  |  |
| *Cryptomys* | 22.3 | 20.1 | 17.7 | 18.4 | - |  |
| *Fukomys* | 21.5 | 21.9 | 19.4 | 18.1 | 17.9 | - |
