# Supplementary material for: Phylogeny and biogeography of the African Bathyergidae: a review of patterns and processes
Source: PeerJ. 2019 Oct 15;7:e7730. doi: 10.7717/peerj.7730 (PMC6798870; doi:10.7717/peerj.7730)
Supplement: Supplemental Information 5 — Pairwise estimates of uncorrected sequence divergence among the various species included and identified within the genus Heliophobius. [file peerj-07-7730-s005.docx]

| **Species** | *H. sp.1* | *H. sp.2* | *H. argenteocinereus* |
| --- | --- | --- | --- |
| *H. sp.1* | - |  |  |
| *H. sp.2* | 13.2 | - |  |
| *H. argenteocinereus* | 12.8 | 9.4 | - |
