# Supplementary material for: Phylogeny and biogeography of the African Bathyergidae: a review of patterns and processes
Source: PeerJ. 2019 Oct 15;7:e7730. doi: 10.7717/peerj.7730 (PMC6798870; doi:10.7717/peerj.7730)
Supplement: Supplemental Information 6 — Pairwise estimates of uncorrected sequence divergence among the various species included and identified within the genus Georychus. [file peerj-07-7730-s006.docx]

| **Species** | *G. sp.1* | *G. sp.2* | *G. sp.3* | *G. sp.4* | *G. capensis* |
| --- | --- | --- | --- | --- | --- |
| *G. sp.1* | - |  |  |  |  |
| *G. sp.2* | 8.9 | - |  |  |  |
| *G. sp.3* | 9.9 | 10.8 | - |  |  |
| *G. sp.4* | 11.8 | 11.7 | 7.9 | - |  |
| *G. capensis* | 11.6 | 12.5 | 7.7 | 6.0 | - |
