# Supplementary material for: Phylogeny and biogeography of the African Bathyergidae: a review of patterns and processes
Source: PeerJ. 2019 Oct 15;7:e7730. doi: 10.7717/peerj.7730 (PMC6798870; doi:10.7717/peerj.7730)
Supplement: Supplemental Information 7 — Pairwise estimates of uncorrected sequence divergence among the various species included and identified within the genus Bathyergus. [file peerj-07-7730-s007.docx]

| **Species** | *B. sp.1* | *B. sp.2* | *B. janetta* | *B. suillus* |
| --- | --- | --- | --- | --- |
| *B. sp.1* | - |  |  |  |
| *B. sp.2* | 3.3 | - |  |  |
| *B. janetta* | 4.6 | 5.3 | - |  |
| *B. suillus* | 4.0 | 4.2 | 3.9 | - |
