# Supplementary material for: Phylogeny and biogeography of the African Bathyergidae: a review of patterns and processes
Source: PeerJ. 2019 Oct 15;7:e7730. doi: 10.7717/peerj.7730 (PMC6798870; doi:10.7717/peerj.7730)
Supplement: Supplemental Information 8 — Pairwise estimates of uncorrected sequence divergence among the various species (subspecies) included and identified within the genus Cryptomys. [file peerj-07-7730-s008.docx]

| **Species** | *C. h. mahali* | *C. h. nimrodi* | *C. h. hottentotus* | *C. h. natalensis* | *C. h. pretoriae* |
| --- | --- | --- | --- | --- | --- |
| *C. h. mahali* | - |  |  |  |  |
| *C. h. nimrodi* | 9.7 | - |  |  |  |
| *C. h. hottentotus* | 10.3 | 8.5 | - |  |  |
| *C. h. natalensis* | 9.1 | 9.5 | 10.3 | - |  |
| *C. h. pretoriae* | 9.5 | 8.3 | 9.3 | 8.1 | - |
