# Supplementary material for: Phylogeny and biogeography of the African Bathyergidae: a review of patterns and processes
Source: PeerJ. 2019 Oct 15;7:e7730. doi: 10.7717/peerj.7730 (PMC6798870; doi:10.7717/peerj.7730)
Supplement: Supplemental Information 9 — Pairwise estimates of uncorrected sequence divergence among the various species included and identified within the genus Fukomys. [file peerj-07-7730-s009.docx]

| **Species** | *F. zechi* | *F. bocagei* | *F. mechowii* | *F. livingstoni* | *F. hanangensis* | *F. amatus* | *F. sp.1* | *F. whytei* | *F. sp.2* | *F. darlingi* | *F. damarensis* | *F. sp.3* | *F. anselli* | *F. micklemi* | *F. choma* | *F. kafuensis* |
| --- | --- | --- | --- | --- | --- | --- | --- | --- | --- | --- | --- | --- | --- | --- | --- | --- |
| *F. zechi* | **-** |  |  |  |  |  |  |  |  |  |  |  |  |  |  |  |
| *F. bocagei* | 16.2 | - |  |  |  |  |  |  |  |  |  |  |  |  |  |  |
| *F. mechowii* | 15.7 | 8.9 | - |  |  |  |  |  |  |  |  |  |  |  |  |  |
| *F. livingstoni* | 14.6 | 10.4 | 9.8 | - |  |  |  |  |  |  |  |  |  |  |  |  |
| *F. hanangensis* | 14.6 | 11.1 | 10.2 | 8.5 | - |  |  |  |  |  |  |  |  |  |  |  |
| *F. amatus* | 15.3 | 10.1 | 9.4 | 8.6 | 7.2 | - |  |  |  |  |  |  |  |  |  |  |
| *F. sp.1* | 15.1 | 10.3 | 9.7 | 9.2 | 7.2 | 4.4 | - |  |  |  |  |  |  |  |  |  |
| *F. whytei* | 15.0 | 10.6 | 10.5 | 9.5 | 6.8 | 5.2 | 5.3 | - |  |  |  |  |  |  |  |  |
| *F. sp.2* | 15.2 | 10.4 | 9.5 | 8.9 | 6.7 | 5.2 | 5.0 | 4.5 | - |  |  |  |  |  |  |  |
| *F. darlingi* | 14.1 | 11.4 | 9.2 | 8.7 | 7.6 | 8.3 | 8.0 | 8.3 | 8.0 | - |  |  |  |  |  |  |
| *F. damarensis* | 14.4 | 9.9 | 10.3 | 8.4 | 7.2 | 7.7 | 8.1 | 7.6 | 7.8 | 7.0 | - |  |  |  |  |  |
| *F. sp.3* | 14.1 | 10.3 | 10.0 | 8.2 | 7.7 | 7.9 | 8.4 | 8.2 | 8.1 | 7.1 | 5.4 | - |  |  |  |  |
| *F. anselli* | 14.4 | 10.5 | 10.2 | 8.6 | 7.8 | 8.6 | 8.3 | 8.5 | 8.4 | 7.0 | 5.6 | 2.7 | - |  |  |  |
| *F. micklemi* | 14.7 | 10.6 | 9.8 | 8.3 | 7.8 | 8.8 | 8.3 | 8.6 | 8.4 | 7.1 | 5.6 | 2.7 | 1.7 | - |  |  |
| *F. choma* | 14.8 | 11.1 | 10.1 | 8.4 | 7.8 | 9.0 | 8.5 | 9.0 | 8.3 | 7.8 | 6.0 | 3.3 | 2.1 | 1.6 | - |  |
| *F. kafuensis* | 14.5 | 10.9 | 9.9 | 8.3 | 7.6 | 8.9 | 8.4 | 8.8 | 8.2 | 6.9 | 5.7 | 3.1 | 2.0 | 1.6 | 1.5 | - |
